# Understanding mechanisms of generalization following locomotor adaptation

**DOI:** 10.1038/s41539-024-00258-2

**Published:** 2024-07-23

**Authors:** Cristina Rossi, Ryan T. Roemmich, Amy J. Bastian

**Affiliations:** 1 Department of Neuroscience, Johns Hopkins University School of Medicine, Baltimore, MD 21205 USA; 2 Center for Movement Studies, Kennedy Krieger Institute, Baltimore, MD 21205 USA; 3 Department of Physical Medicine and Rehabilitation, Johns Hopkins University School of Medicine, Baltimore, MD 21205 USA

**Keywords:** Learning and memory, Motor control

## Abstract

Our nervous system has the remarkable ability to adapt our gait to accommodate changes in our body or surroundings. However, our adapted walking patterns often generalize only partially (or not at all) between different contexts. Here, we sought to understand how the nervous system generalizes adapted gait patterns from one context to another. Through a series of split-belt treadmill walking experiments, we evaluated different mechanistic hypotheses to explain the partial generalization of adapted gait patterns from split-belt treadmill to overground walking. In support of the credit assignment hypothesis, our experiments revealed the central finding that adaptation involves recalibration of two distinct forward models. Recalibration of the first model generalizes to overground walking, suggesting that the model represents the general movement dynamics of our body. On the other hand, recalibration of the second model does not generalize to overground walking, suggesting the model represents dynamics specific to treadmill walking. These findings reveal that there is a predefined portion of forward model recalibration that generalizes across context, leading to overall partial generalization of walking adaptation.

## Introduction

Our nervous system can easily adapt the way we walk to accommodate for changes in our body, like weight gain, or changes in our surroundings, like the ground becoming compliant or squishy after raining^1–6^. Depending on the nature of the change, it may be beneficial or detrimental to maintain the new walking pattern across different contexts—e.g., it would be detrimental to walk indoors as if the ground were compliant, but beneficial to continue accounting for our weight gain both indoors and outdoors. However, the process of switching context is often suboptimal and the new walking pattern is neither fully maintained nor fully abandoned. Rather, adaptation generalizes partially: after adapting in one context, we move differently in other contexts too, although less so than in the original context^7–14^. Why does adaptation generalize partially across contexts, even when this is not optimal and results in errors?

Here, we focused on better understanding the generalization of split-belt treadmill adaptation—where people walk with the feet moving at different speeds—to overground walking. Split-belt treadmill adaptation has been shown to change how people walk overground such that they exhibit aftereffects and limp^15–23^. The aftereffects exhibited overground are similar in nature to those exhibited on a normal-like treadmill (feet moving at the same speed) after adaptation, but they are smaller in magnitude. Overground walking after treadmill adaptation reduces but does not eliminate treadmill aftereffects: even after people revert to walking normally overground, they limp again upon return to a normal-like treadmill, though less so than before overground walking^15,16,20–22^. Understanding how and why treadmill-based adaptation generalizes only partially to overground walking has central importance for translating rehabilitation gains into real-world walking.

We studied how split-belt treadmill adaptation generalizes to overground walking in two experiments. In Experiment 1, we explored the relationship between generalization and individual learning mechanisms that contribute to the overall motor adaptation process. Motor adaptation involves a learning mechanism called “forward model recalibration”^1,4^, and additional learning mechanisms that are task-specific (e.g., reaching adaptation involves “explicit strategies”^24,25^, and walking adaptation involves a “stimulus-response mapping” mechanism that is not under explicit control^26,27^). Here, we focused on studying the generalization of the forward model recalibration mechanism. A “forward model” is an internal representation of our body and/or environment used by our nervous system to predict the sensory outcome of a motor command^1,4^. This forward model is “recalibrated” to re-align predictions about sensory consequences of our movements after accounting for perturbations such as the split-belt treadmill effect. This recalibration alters the way we walk or move, hence contributing to the motor adaptation process. Additionally, this recalibration persists even after the perturbations are removed, resulting in aftereffects such as the observed limping on a normal-like treadmill after split-belt adaptation. Importantly, these treadmill aftereffects result solely from forward model recalibration^26^—movement alterations by other learning mechanisms do not persist after removal of the perturbation^24–26,28^. We speculated that, similar to treadmill aftereffects, overground aftereffects may also result solely from forward model recalibration. As mentioned, overground aftereffects are smaller than treadmill aftereffects^15,16^. Hence, if both types of aftereffects indeed result from the same forward model recalibration mechanism, we would expect that forward model recalibration generalizes partially from the treadmill to the overground context. We tested this hypothesis against the alternative hypotheses that forward model recalibration might either generalize fully or not generalize at all from the treadmill to the overground context.

In Experiment 2, we tested for two alternative hypotheses that could explain why learning acquired through forward model recalibration generalizes partially from treadmill to overground walking (as previously established in Experiment 1). The first hypothesis (the “credit assignment hypothesis”) is that the same recalibration mechanism may update two separate forward models during adaptation: a model representing the general movement dynamics of our body, and a model representing movement dynamics specific to the treadmill walking context^10^. Our nervous system may select which model to adapt by assigning credit for the errors experienced in split-belt walking to either our body or the treadmill; only adaptations to the model of our body would generalize to overground walking^10,15,16,19,29,30^. In support of this idea, adapting from small, gradual errors results in larger generalization than large, abrupt errors, probably because small errors are closer to natural walking variability and are more likely to be attributed to one own’s body^16,29^. Furthermore, credit assignment is thought to underly generalization of reaching adaptation from a robotic device to natural free-space movement^10,31^.

The second hypothesis (the “slow switching hypothesis”) is that the partial generalization of adaptation across contexts reflects a delay in switching between context-specific walking patterns^15,21–23,32–34^. For example, even if we only recalibrated a forward model specific to the treadmill context, and not a general model of our body dynamics, we may exhibit aftereffects overground because our nervous system may be slow at transitioning from treadmill-specific to overground-specific control of walking. Indeed, generalization has been found to increase if the visual or proprioceptive cues that mediate awareness of context-switching are limited^15,19^. This led to the idea that generalization may reflect the temporary failure to retrieve the correct walking pattern from memory^17^, because memory retrieval is known to be imperfect and modulated by contextual cues^35^. Indeed, cues can help with retrieving the appropriate calibration in reaching adaptation tasks^36,37^. Furthermore, recent work has shown that in older ages—when motor switching processes are in decline—overground transfer tends to be larger^22,23,32^, is related to cognitive switching abilities, and is reduced by previous practice with switching between treadmill and overground contexts^23^. Given this strong evidence, we predicted that we would see evidence for slow switching between treadmill and overground walking patterns, rather than for credit assignment.

## Results

### Experiment 1A: partial generalization of forward model recalibration learning

The goal of Experiment 1A was to test our hypothesis that learning acquired through forward model recalibration generalizes partially from the treadmill to overground, contrasted with the alternative hypotheses proposing full or no generalization.

#### Motor generalization is consistent with previous work

**Fig. 1:**
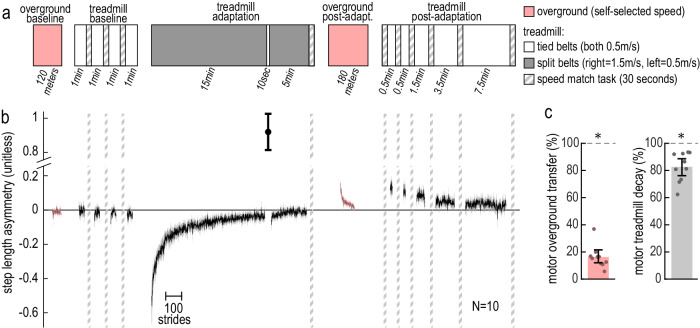
Experiment 1A, replication of motor results. **a** Experimental protocol. Pink boxes indicate blocks of overground walking, grayscale boxes indicate blocks of treadmill walking on tied-belts (white), split-belts (solid gray) or speed match tasks (dashed gray). **b** Step length asymmetry time course (group mean ± SE), position and color of each block match that of the protocol shown in (**a**). The length of each block is truncated to the participant with the fewest strides. **c** Measures of motor generalization: motor overground transfer and motor treadmill decay. Bars and error bars depict group mean ± CI, and dots depict individual participants. Asterisks indicate that the measure is significantly greater than 0% and lesser than 100%.

We studied walking adaptation and generalization using a standard protocol (Fig. 1a) and a well-established measure for movement error during walking, step length asymmetry (difference between right and left step lengths, normalized by their sum; Figs. 1b and S1a)^15–18,20–23,38,39^. In baseline, participants walked with close-to-zero step length asymmetry both overground and on treadmill tied-belts (pink and black traces in Fig. 1b; step length asymmetry in overground baseline = −0.0110 [−0.0222, 0.0001], treadmill baseline = −0.0145 [−0.0416, 0.0116], mean [CI]; Supplementary Fig. 2a). Participants adapted on the treadmill with split-belts for 20 min (right belt at 1.5 m/s, left belt at 0.5 m/s), showing a typical step length asymmetry learning curve (initially negative and gradually reapproaches zero). We measured treadmill motor aftereffects *before* overground washout with a brief tied-belt catch trial after 15 min of adaptation. Participants exhibited significantly positive step length asymmetry in the catch trial (step length asymmetry in catch trial = 0.9203 [0.7254, 1.1590], mean [CI]; Supplementary Fig. 2a), which is indicative of treadmill aftereffects. As expected, step length asymmetry also appeared positive at the start of the “overground post-adaptation” block (pink in Fig. 1a, b), but *less* positive than in the treadmill catch trial. To quantify this, we computed “motor overground transfer”—one measure of generalization of motor learning (baseline-normalized ratio of overground to treadmill aftereffects, see “Methods”). Indeed, we found that the motor overground transfer was significantly greater than zero and significantly lesser than 100% (motor overground transfer = 16.26% [12.04, 21.69]%, mean [CI]; Fig. 1c). This confirms the presence of motor aftereffects overground that are smaller than the treadmill aftereffects. Participants then returned to the treadmill and walked on tied-belts during “treadmill post-adaptation”. As expected, treadmill aftereffects were still present (significantly positive step length asymmetry) but decreased by ~80% as compared to those measured by the catch trial due to overground washout. To confirm this, we computed the “motor treadmill decay”, another measure of motor generalization (see “Methods”), and found that it was significantly greater than zero and significantly lesser than 100% (motor treadmill decay = 82.67% [76.07, 88.74]%, mean [CI]; Fig. 1c). In summary, aftereffects decreased in magnitude from treadmill to overground walking, and then further from overground walking back to treadmill walking. These findings are consistent with a large body of work^15–18,20–23^.

#### Forward model recalibration is measured using perceptual tests

As mentioned, the main hypothesis of Experiment 1A was that learning acquired through forward model recalibration generalizes partially from the treadmill to overground. The forward model recalibration mechanism can be dissected and measured using tests of leg speed perception^26^. This is because forward model recalibration alters not only the way we *move*, but also the way we *perceive* our movements^40,41^. During split-belt adaptation, the perception of leg speed is gradually altered so that the leg speeds feel *less* asymmetric than they truly are^26,42–44^. This perceptual alteration results solely from forward model recalibration, because other learning mechanisms involved in motor adaptation alter movement but not perception^26,45,46^.

**Fig. 2:**
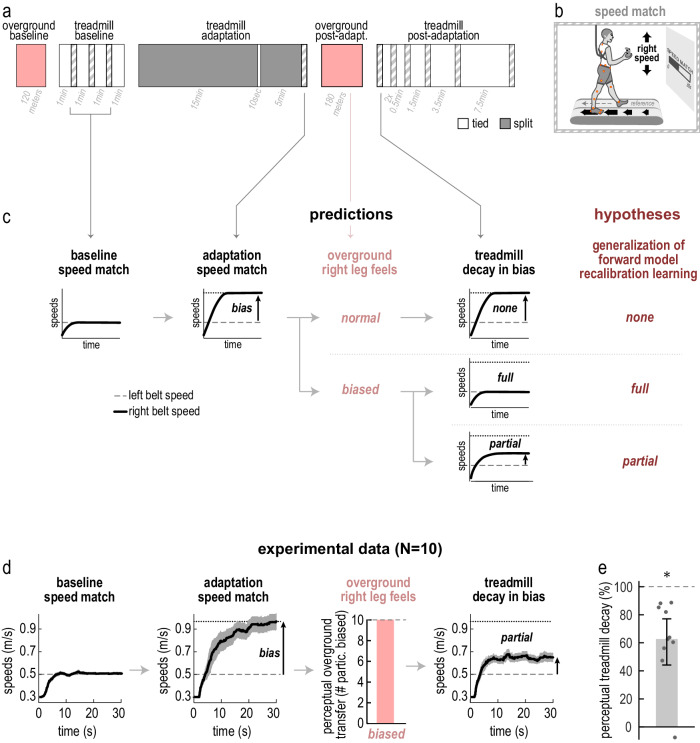
Experiment 1A, generalization of recalibration. **a** Experimental protocol (as in Fig. 1a). **b** Speed match task setup. Participants controlled the right belt speed and tried to match it the left “reference” speed. **c** Predictions for perceptual results made by three competing hypotheses: generalization of forward model recalibration is (1) none (top row), (2) full (middle row), or (3) partial (bottom row). “baseline speed match”: schematic time course of the left (dashed gray) and right (solid black) belt speed during a baseline speed match task. Participants accurately match the right speed to the left speed. “adaptation speed match”: schematic for the task performed at the end of adaptation. Participants overshoot the right speed. We compute bias as right minus left speed at the end of the task. Baseline and adaptation predictions are based on previous work and do not differ across hypotheses. “overground right leg feels”: prediction for the overground post-adaptation questionnaire. Perceptual bias is predicted by the “full” and “partial” generalization hypotheses, but not the “none” hypothesis. “treadmill decay in bias”: prediction for the first speed match task in treadmill post-adaptation. The (1) “none”, (2) “full” and (3) “partial” generalization hypotheses respectively predict (1) same perceptual bias as adaptation speed match (decay is none), (2) no bias (full decay), or (3) smaller bias (partial decay). **d** Experimental results for the predictions in (**c**). Solid black line with solid gray shade depicts right speed (group mean ± SE); dashed gray line depicts left speed. “baseline speed match” (average of three iterations) and “adaptation speed match” are consistent with the predictions in (**c**). “overground right leg feels”: all ten participants reported a perceptual bias at the start of overground post-adaptation (pink bar), consistent with the “full” or “partial” generalization hypotheses. “treadmill decay in bias”: speeds in the first post-adaptation speed match task. The bias is less than the adaptation speed match task, consistent with the “partial” generalization hypothesis. **e** Perceptual treadmill decay measure (bar ± error bar = group mean ± CI, dots = individual participants, asterisk indicates significant difference from 0 and 100%).

We measure leg speed perception using a “speed match task”, where people select belt speeds that feel equal by changing the speed of the right treadmill belt with a joystick^26^ (Fig. 2a, b). The two leftmost panels in Fig. 2c schematize belt speeds typically observed in speed match tasks^26,42–44^. In baseline (“baseline speed match”), people accurately match the belt speeds (i.e., they change the speed of the right belt until it matches the left). After adaptation (“adaptation speed match”), people overshoot the speed of the right belt so that it moves faster than the left. This is because they have developed a perceptual bias where the right leg feels slower than its true speed. We compute perceptual bias as the belt speed difference at the end of the task (“bias” = right–left speed). It has been shown that the perceptual bias provides a robust measure of the changes to the walking pattern that arise specifically from the recalibrated forward model^26,40^, isolating the effect of forward model recalibration from that of other mechanisms.

We therefore study generalization of the forward model recalibration mechanism by evaluating measures of generalization of the perceptual bias: “perceptual overground transfer” and “perceptual treadmill decay”. We tested perceptual overground transfer using a questionnaire, where we asked participants whether their right leg felt biased (“not as expected”) during overground post-adaptation (because the speed match task cannot be performed overground). We tested perceptual treadmill decay by performing another iteration of the speed match task after overground post-adaptation (Fig. 2a, first speed match task in treadmill post-adaptation). We compared the perceptual bias in this task to that measured by the adaptation speed match task: any decay in perceptual bias between these tasks would be due to washout occurring in the overground post-adaptation block.

#### Partial perceptual treadmill decay supports partial generalization of forward model recalibration

We illustrate relevant predictions made by the different hypotheses in Fig. 2c. The top row illustrates the alternative hypothesis that forward model recalibration may not generalize at all to overground walking—meaning it would neither influence the walking pattern nor wash out overground. This hypothesis predicts no perceptual overground transfer (the right leg would not feel biased in overground post-adaptation) and no perceptual treadmill decay (the perceptual bias on the treadmill would be the same before and after overground post-adaptation). The middle row illustrates the alternative hypothesis that forward model recalibration may generalize completely to overground walking—meaning it would influence the walking pattern and fully wash out overground. This hypothesis predicts perceptual overground transfer—the right leg would feel biased in overground post-adaptation—and full perceptual treadmill decay—there would be no perceptual bias on the treadmill after overground post-adaptation. The bottom row illustrates the main hypothesis that forward model recalibration generalizes partially to overground walking—meaning it would influence the walking pattern and partially wash out overground. This hypothesis predicts perceptual overground transfer and partial perceptual treadmill decay—the perceptual bias on the treadmill would be smaller after overground post-adaptation.

Figure 2d shows experimental results paralleling the predictions illustrated in Fig. 2c (other speed match tasks are shown in Supplementary Fig. 2c; individual data is shown in Supplementary Fig. 1b). Consistent with previous work, the perceptual bias was not significant in the baseline speed match tasks (baseline perceptual bias = 0.0078 [−0.0145, 0.0320] m/s, mean [CI]; Supplementary Fig. 2b), but was significant in the adaptation speed match task (adaptation perceptual bias = 0.4665 [0.3060, 0.6080] m/s, mean [CI]; Supplementary Fig. 2b). We found that all participants reported a perceptual bias (right leg initially felt “not as expected”) in overground post-adaptation—signifying perceptual overground transfer (the perceptual bias decayed by the end of the overground post-adaptation block in all but two participants). The right panel in Fig. 2d shows the first speed match task in treadmill post-adaptation, performed right after overground post-adaptation. There appeared to be a perceptual bias in this task that was smaller than the bias observed in the adaptation speed match task, suggesting a partial decay in perceptual bias. We quantified this with the “perceptual treadmill decay” measure, depicted in Fig. 2e. Indeed, we found that the perceptual treadmill decay was significantly larger than 0% and significantly smaller than 100% (perceptual treadmill decay = 62.69% [44.13, 77.10]%, mean [CI]). These results confirm our hypothesis that forward model recalibration generalizes *partially* to overground walking.

### Supplementary analysis of different gait kinematic parameters

We performed a supplementary analysis to investigate generalization of adapted gait kinematic parameters other than step length asymmetry. We examined joint kinematic patterns, limb configuration parameters, and temporal parameters of the walking pattern. This is described in detail in Supplementary Note 1, “Analysis of different gait kinematic parameters” (“Results”, “Discussion”, “Methods”), Supplementary Figs. 3, 4 and Supplementary Tables 1–3. Importantly, the analysis demonstrated that treadmill adaptation alters both knee and hip joint angles in overground walking, offering key insights for the development of gait rehabilitation interventions.

### Experiment 1B: partial generalization of forward model recalibration is robust to overground walking distance and speed

#### Long Preferred and Short Slow walk overground for a longer distance or at a slower speed

The goal of Experiment 1B was to ensure that our interpretation that forward model recalibration generalizes partially to overground walking was robust to potential confounds. We considered that there may be two alternative explanations for why the perceptual bias decayed partially and not fully during overground post-adaptation. First, the post-adaptation overground block may have been too short and participants may have needed to walk for longer in order to fully washout. Second, the overground walking speed may have limited the washout extent. It is known that washing out at one speed does not completely eliminate aftereffects at a different speed, at least when both conditions are on the treadmill^47^. Therefore, it is possible that washing out in overground post-adaptation did not completely eliminate treadmill aftereffects because of the walking speeds differing between the two blocks.

To this end, we aimed to replicate the results of Experiment 1A in two groups that, in overground post-adaptation, walked (1) for a longer distance, and (2) at a speed matching the treadmill post-adaptation speed. The “Long Preferred” group walked for 480 meters, in contrast to the 180 meters walked by the group of Experiment 1A—which we will refer to as “Short Preferred”. The “Short Slow” group walked at ~0.5 m/s, i.e., the treadmill post-adaptation speed, in contrast to the self-selected speed of Short and Long Preferred. This was achieved by informing participants whether they walked at the desired speed between 0.45–0.55 m/s or too fast or slow after each overground pass (see “Methods”). The paradigms for the Short Preferred, Long Preferred and Short Slow groups were otherwise identical.

We first ensured that participants performed the distance and speed manipulations as desired (these confirmatory results, as well as step length asymmetry and bias time courses, are shown in Supplementary Figs. 5 and 6). We confirmed that the Long Preferred group took more strides than the Short Preferred group in overground post-adaptation overground but not in baseline (difference in number of strides between Long and Short Preferred, post-adaptation = 140.80 [116.90, 165.80] strides, baseline = −6.80 [−16.80, 2.40] strides, mean [CI]; Supplementary Fig. 6a, c). The walking speed did not significantly differ between Short and Long Preferred (difference in walking speed between Long and Short Preferred, baseline = 0.0226 [−0.0894, 0.1391] m/s, post-adaptation = 0.0652 [−0.0461, 0.1782] m/s, mean [CI]; Supplementary Fig. 6b, d). We also verified that the overground walking speeds of the Short Slow group were not significantly different from 0.5 m/s at the group level (walking speed in baseline = 0.4981 [0.4778, 0.5193] m/s, post-adaptation = 0.4986 [0.4807, 0.5161] m/s, mean [CI]) and were significantly slower than those of the Short Preferred group (difference in walking speed between Short Slow and Short Preferred, baseline = −0.5439 [−0.6337, −0.4475] m/s, post-adaptation = −0.5122 [−0.5875, −0.4271] m/s, mean [CI]). The walking speeds for all individual participants in the Short Slow group did not significantly differ from 0.45–0.55 m/s (all CI_LB_ < 0.51 m/s and all CI_UB_ > 0.46 m/s; Supplementary Table 4 and Supplementary Fig. 7), in contrast to all participants in the Short and Long Preferred groups who walked significantly faster (all CI_LB_ > 0.63 m/s; Supplementary Table 4 and Supplementary Fig. 7). As expected given that strides are shorter for slower speeds^48^, the Short Slow group took more strides overground than the Short Preferred group (difference in number of strides between Short Slow and Short Preferred, baseline = 39.20 [26.50, 51.20] strides, post-adaptation = 65.80 [47.60, 83.20] strides, mean [CI]; Supplementary Fig. 6a, c).

#### Generalization is not affected by overground walking distance or speed

**Fig. 3:**
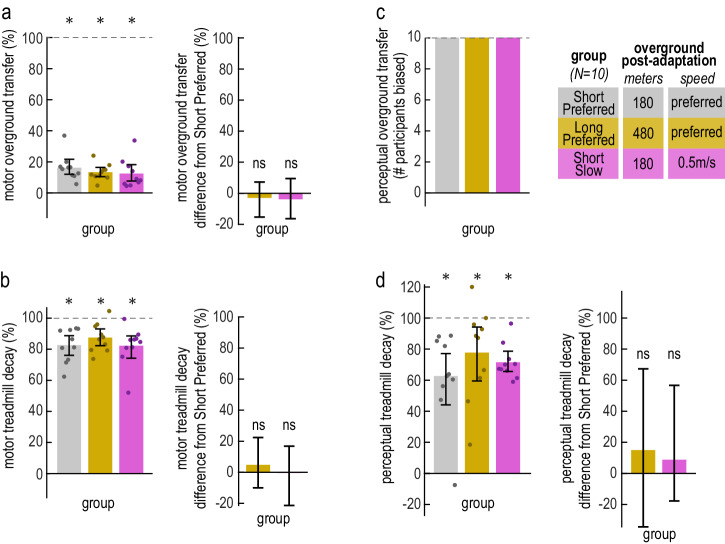
Experiment 1B, robustness of generalization to speed and distance. Motor and perceptual generalization results for the three groups: Short Preferred (gray, same data as Experiment 1A, Figs. 1 and 2), Long Preferred (yellow), and Short Slow (pink). In (**a**), (**b**), and (**d**), bars and error bars depict group mean ± CI, dots depict individual participants, and asterisks indicate that the measure is significantly different from 0 and 100% (within-group analyses), or significantly different between the groups. **a** Left: motor overground transfer measure for each group. Right: difference in motor overground transfer between Long Preferred and Short Preferred (yellow), and between Short Slow and Short Preferred (pink). **b** Motor treadmill decay measure and difference between groups, organized like (**a**). **c** Perceptual overground transfer measure, i.e., number of participants in each group with a perceptual bias at the start of overground post-adaptation. **d** Perceptual treadmill decay measure and difference between groups, organized like (**a**) and (**b**).

We then asked whether the distance or speed manipulations affected motor or perceptual generalization from the treadmill to overground walking. Consistent with Experiment 1A, we found *partial* motor generalization for both Long Preferred and Short Slow groups, using either motor overground transfer or motor treadmill decay measures (Long Preferred motor overground transfer = 13.31% [10.45, 16.42]%, motor treadmill decay = 87.46% [82.26, 93.05]%, Short Slow motor overground transfer = 12.43% [7.62, 18.22]%, motor treadmill decay = 82.21% [74.27, 88.57]%, mean [CI]; Fig. 3a, b). Furthermore, motor generalization was not significantly different between these groups and Short Preferred (motor overground transfer difference between Long and Short Preferred = −2.95% [−8.95, 2.44]%, between Short Slow and Short Preferred = −3.83% [−11.14, 3.44]%, motor treadmill decay difference between Long and Short Preferred = 4.79% [−3.22, 13.41]%, between Short Slow and Short Preferred = −0.46% [−10.27, 8.87]%, mean [CI]; Fig. 3a, b). Like the Short Preferred group, all participants in the Long Preferred and Short Slow groups reported a perceptual bias in overground post-adaptation, signifying perceptual overground transfer (Fig. 3c; the bias dissipated in most participants by the end of the block, Supplementary Fig. 6e). Also like Short Preferred, the perceptual treadmill decay was *partial* for both Long Preferred and Short Slow groups—it was significantly greater than zero and less than 100% (perceptual treadmill decay for Long Preferred = 77.72% [59.48, 94.17]%, Short Slow = 71.52% [65.63, 78.66]%, mean [CI]; Fig. 3d). The perceptual treadmill decay was not significantly different between these groups and Short Preferred (perceptual treadmill decay difference between Long and Short Preferred = 15.02% [−8.65, 39.95]%, between Short Slow and Short Preferred = 8.82% [−7.20, 28.43]%, mean [CI]; Fig. 3d).

In sum, in Experiment 1B we replicated the results of Experiment 1A for different walking speed and distance traveled in overground post-adaptation, corroborating the finding that the forward model recalibration mechanism generalizes *partially* to overground walking.

### Experiment 2A: credit assignment—not slow context switching—underlies partial generalization

In Experiment 2A, we evaluated two alternative hypotheses for the partial generalization of forward model recalibration from the treadmill to overground walking: credit assignment and slow switching.

#### Hypotheses are dissociated with repeated treadmill-overground switching

**Fig. 4:**
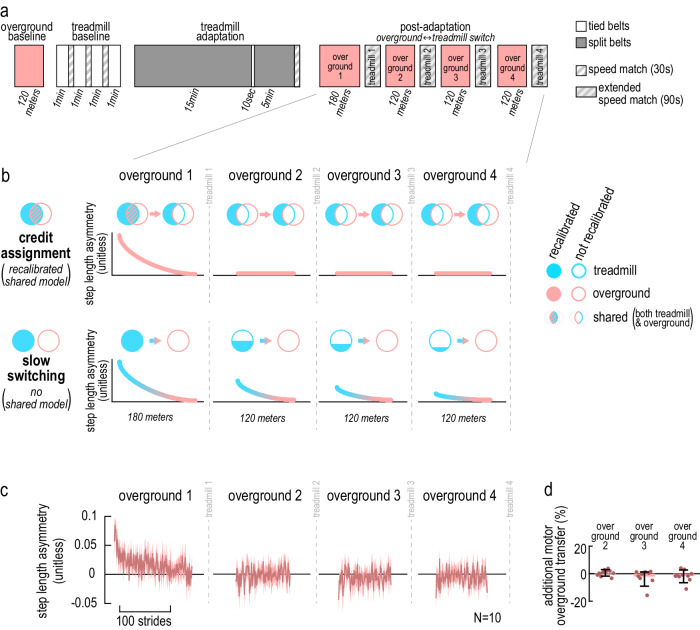
Experiment 2A, evaluation of credit assignment versus slow switching. **a** Experimental protocol. Pink: overground walking, white: tied-belts, solid gray: split-belts, dashed gray: speed match tasks (narrower, baseline and adaptation) or extended speed match tasks (wider, post-adaptation). **b** Predictions for step length asymmetry in overground post-adaptation made by two competing hypotheses. Credit assignment (top): treadmill and overground walking patterns are partially separate and partially shared (blue and pink circles partially overlap). In adaptation, treadmill and shared models are recalibrated (filled blue circle, hatched overlap of blue-pink circles). “Overground 1”: initial positive step length asymmetry reflects recalibration of the shared model (hatched blue-pink overlap). Step length asymmetry returns to zero as the shared model recalibration washes out (blue-pink overlap becomes not filled). “Overground 2–4”: Step length asymmetry remains zero, because the shared model is not recalibrated (blue-pink overlap is not filled). Slow switching (bottom): treadmill and overground walking patterns are fully separate (blue and pink circles do not overlap). In adaptation, the treadmill model is recalibrated (filled blue circle). “Overground 1”: initial positive step length asymmetry (blue portion of the line) reflects recalibration of treadmill model (filled blue circle). Step length asymmetry returns to zero (pink portion of the line) as the control of walking gradually transitions to the overground model, which is not recalibrated (pink circle, not filled). “Overground 2–4”: Step length asymmetry is initially positive again (blue portion of the line) because the treadmill model is still partially recalibrated (partially filled blue circle) and temporarily controls walking again after each treadmill-to-overground switch. **c** Experimental results: step length asymmetry in overground post-adaptation (group mean ± SE; each block is truncated in length to match the participant with fewest strides). Step length asymmetry appears to remain zero in “Overground 2–4”, in line with the prediction from the credit assignment hypothesis. **d** Additional motor overground transfer for “Overground 2–4”, i.e., normalized step length asymmetry at the start of each block relative to the end of the previous block. Bars and error bars depict group mean ± CI, dots depict individual participants. CIs overlap zero, indicating no significant difference from zero.

We used the switch paradigm depicted in Fig. 4a to dissociate these hypotheses. The baseline and adaptation phases were the same as in Experiment 1, but here participants repeatedly switched between treadmill and overground contexts in post-adaptation. We specifically tested four post-adaptation overground blocks interleaved with “extended speed match task” treadmill blocks. The extended speed match task consisted of a speed match task (described in Experiment 1) followed by 90 seconds of walking with belt speeds that felt equal. This manipulation eliminated the sensory prediction error thought to drive recalibration^26,42,44,49^, so that we could study the effect of switching context without re-adaptation or washout of the forward models during the treadmill blocks.

Figure 4b illustrates the theoretical framework and data predictions for both hypotheses. According to both hypotheses, our nervous system employs distinct forward models to control walking in different contexts: a model for treadmill walking (abbreviated as “treadmill model”; blue circles), and a model for overground walking (abbreviated as “overground model”; pink circles). In the framework proposed by the credit assignment hypothesis, an additional “shared model” is employed to control of walking in both contexts: both the treadmill model and the shared model are recalibrated during adaptation (top row in Fig. 4b, blue-pink circles overlap; note that this is the forward model of our body dynamics described in the introduction). In contrast, the framework proposed by the slow switching hypothesis does not incorporate the idea of a shared model: only the treadmill model is recalibrated during adaptation (bottom row in Fig. 4b).

The hypotheses provide distinct explanations for the step length asymmetry aftereffects observed in the “overground 1” block (which corresponds to the overground post-adaptation block of Experiment 1). They also make different predictions regarding the presence of step length asymmetry aftereffects in subsequent overground post-adaptation.

#### “Credit assignment” predicts no aftereffects in post-adaptation overground blocks 2–4

The credit assignment hypothesis suggests that the step length asymmetry aftereffects in “overground 1” result from the recalibration of the shared model (Fig. 4b top, recalibrated blue-pink dashed circle underlies the positive step length asymmetry). The shared model is employed in both contexts, so that it is recalibrated during treadmill adaptation, and this recalibration then influences overground walking leading to overground aftereffects. The recalibration of the shared model washes out in “overground 1” as people readjust to walking without perturbations, so that step length asymmetry reverts to zero (Fig. 4b top, blue-pink circles overlap is not recalibrated at the end of “overground 1”). The shared model remains not recalibrated in subsequent overground blocks. Consequently, the credit assignment hypothesis predicts no step length asymmetry aftereffects (i.e., step length asymmetry close to zero) in the “overground 2”, “overground 3”, and “overground 4” blocks.

#### “Slow switching” predicts aftereffects in all post-adaptation overground blocks

In contrast, the slow switching hypothesis suggests that the step length asymmetry aftereffects in “overground 1” result from the recalibration of the treadmill model (Fig. 4b bottom, recalibrated blue circle underlies the positive step length asymmetry). According to this hypothesis, the nervous system aims to control walking in the treadmill versus overground contexts using distinct models—the treadmill model and the overground model. However, it is unable to switch between these models immediately. As we transition from the treadmill context of “treadmill adaptation” to the overground context of “overground 1”, the nervous system temporarily persists in employing the treadmill model to control walking. This results in overground aftereffects in step length asymmetry, even though the overground model has not been recalibrated. The nervous system switches to the overground model gradually throughout “overground 1”, and step length asymmetry reverts to zero (Fig. 4b bottom, pink circle is not filled). Importantly, although the treadmill model no longer influences the walking pattern at the end of “overground 1”, it remains recalibrated (at least partially—some washout is expected before the model switch). Upon transitioning to the treadmill context of “treadmill 1”, the treadmill model once again controls the walking pattern. Similar to “overground 1”, when we transition back to the overground context in “overground 2”, control by the treadmill model temporarily persists, resulting in step length asymmetry aftereffects (Fig. 4b bottom, partially recalibrated blue circle underlies the positive step length asymmetry). A similar process would repeat for the remaining post-adaptation blocks—hence, the slow switching hypothesis predicts positive step length asymmetry aftereffects at the start of “overground 2”, “overground 3”, and “overground 4” blocks.

#### Absence of step length asymmetry aftereffects in blocks 2–4 supports credit assignment

Figure 4c shows step length asymmetry data in the post-adaptation overground blocks for participants that underwent the switch paradigm (other time points, as well as generalization measures, are reported in Supplementary Note 2, “Control analyses for Experiment 2” and Supplementary Fig. 8; individual data are shown in Supplementary Fig. 9). Participants appeared to only exhibit aftereffects in “overground 1”, while step length asymmetry seemed to remain close to zero for blocks “overground 2”, “3”, and “4”. To evaluate this, for each of the latter three blocks we computed the measure of “additional motor overground transfer”—step length asymmetry at the start of a block minus the end of the previous block, baseline-subtracted and normalized to the treadmill catch (see “Methods”). We found that this measure was not significantly different from zero for any of the blocks (additional motor overground transfer for “overground 2” = 0.58% [−0.75, 1.84]%, for “overground 3” = −2.08% [−5.52, 0.44]%, for “overground 4” = −1.56% [−4.16, 0.71]%, mean [CI]; Fig. 4d). This confirms that there are no step length asymmetry aftereffects in the “overground 2”, “overground 3”, and “overground 4” blocks. These results contrast with predictions from the slow switching hypothesis and instead support the credit assignment hypothesis.

### Experiment 2B: treadmill aftereffects are unaffected by further overground walking, confirming the credit assignment hypothesis

#### Reference group does not walk in overground post-adaptation blocks 2–4 to assess unwanted washout effects

In Experiment 2B we performed an additional test of the competing credit assignment and slow switching hypotheses to rule out potential confounds in our interpretation. We considered the possibility that, despite using the extended speed match task for the post-adaptation treadmill blocks, there may still be some unwanted washout of the forward models recalibrations by factors unrelated to overground walking (e.g., forgetting/unlearning due to passage of time, getting off/on the treadmill, and/or the slow right speed at the start of the speed match task). We therefore aimed to exclude the possibility that overground aftereffects may have been present in the post-adaptation blocks “overground 2”, “3”, and “4” of Experiment 2A, but masked by unwanted washout effects.

**Fig. 5:**
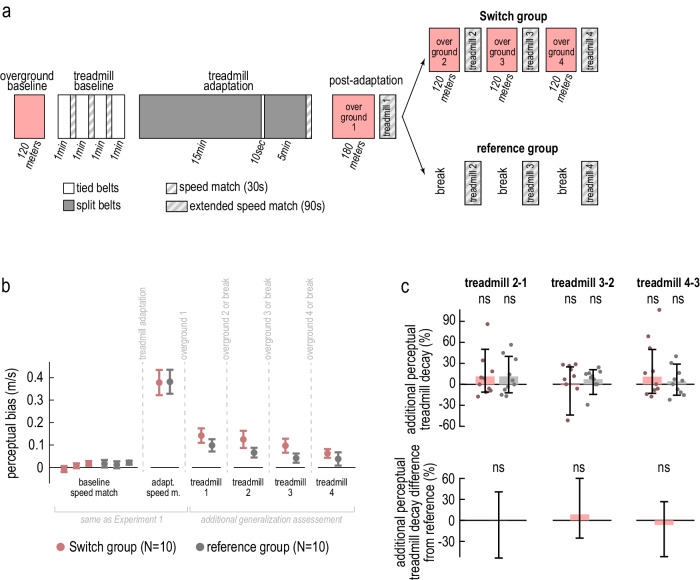
Experiment 2B, validation of credit assignment hypothesis. **a** Experimental protocols for the Switch group (top, same as Fig. 4a), and reference group (bottom, taking sitting breaks in place of “overground 2”, “3”, and “4”). **b** Perceptual bias for speed match tasks in baseline and adaptation (same as Experiment 1), and for extended speed match tasks in post-adaptation (iterations used to compute the additional treadmill decay measure in (**c**)), for the Switch (pink) and reference (gray) groups (group mean ± SE). **c** Additional perceptual treadmill decay for each group (top), and difference between the groups (bottom). Bars and error bars depict group mean ± CI, dots depict individual participants, and “ns” indicates the measure is not significantly different from zero (top) or between groups (bottom).

We collected an additional “reference” group of participants who did not walk for the post-adaptation blocks “overground 2”, “3”, and “4”. Instead, they took sitting breaks, off the treadmill, that matched the duration of walking for the group of Experiment 2A—here referred to as “Switch” (Fig. 5a). The remainder of the paradigm was identical between the groups. We computed the measure of “perceptual bias” for baseline, adaptation, and each of the post-adaptation treadmill blocks - “treadmill 1”, “2”, “3”, and “4”—using the respective (extended) speed match tasks (Fig. 5b; see also Supplementary Note 2, “Control analyses for Experiment 2”, and Supplementary Figs. 8 and 9).

#### Slow switching, but not credit assignment, predicts additional perceptual treadmill decay with overground walking

The “perceptual bias” measure captures the recalibration of the “treadmill model” discussed in Experiment 2A (blue circles, Fig. 4b). In the slow switching hypothesis framework, the treadmill model recalibration decays during post-adaptation blocks “overground 2”, “3”, and “4” of the Switch group, because the treadmill model is temporarily used to control overground walking in these blocks. As a result, the treadmill perceptual bias would decay across post-adaptation treadmill blocks, and the effect would be tied to overground walking. In contrast, in the credit assignment hypothesis framework, there is no decay in perceptual bias caused by overground walking. We isolated the effect of overground walking by comparing the decay in perceptual bias between Switch and reference groups—the latter did not walk overground, so that any decay would be related to unwanted factors. The slow switching hypothesis predicts that the perceptual bias would decay faster for the Switch group than the reference group. The credit assignment hypothesis predicts that the perceptual bias would either not decay, or decay similarly between the groups (due to factors unrelated to overground walking).

#### No additional perceptual treadmill decay supports credit assignment

We first computed the measure of “additional perceptual treadmill decay” for each pair of consecutive post-adaptation treadmill blocks (“treadmill 2-1”, “treadmill 3-2”, “treadmill 4-3”)—i.e., the normalized difference in perceptual bias between the pair of blocks (Fig. 5c, top). There appeared to be a slight decay in perceptual bias in both groups; however, the decay was not statistically different from zero for any of the blocks (“additional perceptual treadmill decay” for the Switch group for “treadmill 2-1” = 11.54% [−3.72, 31.17]%, “treadmill 3-2” = −1.30% [−24.33, 17.78]%, “treadmill 4-3” = 10.78% [−4.53, 28.87]%, for the reference group for “treadmill 2-1” = 11.59% [−3.03, 27.42]%, “treadmill 3-2” = 7.19% [−2.50, 14.80]%, “treadmill 4-3” = 4.28% [−6.97, 16.29]%, mean [CI]). We then computed the difference in “additional perceptual treadmill decay” between groups (Fig. 5c, bottom). As expected, this measure was not different from zero for any of the pairs of treadmill blocks (difference in “additional perceptual treadmill decay” between Switch and reference groups for “treadmill 2-1” = −0.05% [−22.72, 24.09]%, “treadmill 3-2” = −8.49% [−32.91, 12.78]%, “treadmill 4-3” = 6.51% [−13.23, 27.99]%, mean [CI]). This supports the credit assignment hypothesis, confirming results from Experiment 2A.

In sum, in Experiment 2 we show that repeatedly switching between treadmill and overground walking contexts after overground washout does not lead to further generalization, despite the lingering treadmill aftereffects. Our findings support the credit assignment hypothesis—i.e., that adaptation generalizes partially to overground walking because it recalibrates not only a treadmill model but also a shared model (shared by overground and treadmill walking contexts).

## Discussion

In this study, we aimed to explain the partial generalization of split-belt adaptation from the treadmill to overground walking. Our results suggest that adaptation involves recalibration of both (1) forward models of our body that generalize to overground walking, and (2) forward models specific to treadmill walking that do not generalize. More specifically, our findings reveal that forward model recalibration generalizes partially to overground walking. They also demonstrate that switching between treadmill and overground walking patterns is not a slow process in itself: after the first block of overground washout, people switch walking pattern immediately as they get off the treadmill. Instead, we find that there is a predefined portion of the adapted walking pattern that generalizes across contexts, and another portion that is treadmill specific, leading to overall partial generalization.

We found that the bias in leg speed perception developed with split-belt adaptation generalizes partially to overground walking. Consistent with previous work, participants exhibited a perceptual bias of ~0.4 m/s immediately after adaptation (right leg feels ~0.4 m/s slower than its true speed)^42–44,50^. Here, we show that the perceptual bias transfers to overground walking and decays partially with overground washout: upon return to the treadmill, the bias was ~0.1 m/s, which was significantly larger than baseline and significantly smaller than post-adaptation. We recently demonstrated that split-belt adaptation involves two types of learning mechanisms—forward model recalibration and another mechanism akin to stimulus-response mapping—and that the perceptual bias is a reliable measure of forward model recalibration specifically^26,40^. Hence, our results indicate that forward model recalibration generalizes partially from the treadmill to overground walking.

These results are consistent with previous observations that post-adaptation aftereffects on the treadmill are attributed to forward model recalibration alone (other mechanisms of adaptation such as stimulus-response mappings are not thought to contribute to aftereffects)^1,24,26,28^. Based on this framework, we hypothesized that forward model recalibration may also be the sole contributor to aftereffects overground. Our findings are consistent with this suggestion, because the partial generalization of recalibration may directly result in the aftereffects being smaller overground than on the treadmill, as observed here and in previous work^15–18,20–23^.

The idea that forward model recalibration plays a key role in generalization is consistent with a large body of work. Mariscal et al. found that overground transfer increases when participants’ attention is altered during split-belt adaptation^20^—a manipulation which may increase the relative contribution of forward model recalibration to adaptation^51–53^. Similarly, work in reaching adaptation suggests that forward model recalibration accounts for generalization of aftereffects across different task conditions^54–56^, limbs^57–59^, and to natural settings^8^.

Contrary to our predictions, our results are at odds with the slow switching hypothesis. In fact, after the first block of overground washout, people can immediately switch from walking on the treadmill with an adapted walking pattern, to walking overground with a “normal”, not adapted walking pattern. This demonstrates that overground aftereffects consist of a specific, predefined portion of the treadmill aftereffects. Once this portion has been washed out overground, getting on and off the treadmill again does not result in further aftereffects overground, even though treadmill aftereffects are still present. Our findings are consistent with the credit assignment hypothesis, suggesting that during split-belt adaptation we recalibrate both a forward model of our body—that generalizes across contexts—and a forward model for treadmill walking^10,60,61^—that does not generalize. The hypothesis is also supported by previous studies of reaching adaptation in response to robotic force perturbations, which show that people attribute part of the perturbation to their arm and part to the robot, recalibrating both forward models^31,62^. Similarly, the sensory prediction error experienced on the split-belt treadmill may be partially attributed to the legs, and partially to the treadmill^15,16,29^. The recalibration of the forward model of our legs may transfer to overground walking because we do not expect our legs to be any different in this context, while the recalibration of the forward model for treadmill walking may be abandoned in a different context. In line with this idea, generalization is larger when the split-belt perturbation is introduced gradually rather than abruptly, likely because the smaller errors feel more natural and are attributed to the body to a greater extent^16,29^.

We propose that the modular architecture suggested to underly motor control and learning^63–65^ may help reconcile our findings with previous work that had instead appeared to support the slow switching hypothesis. Overground generalization has been shown to increase when vision is occluded^15^ or proprioceptive noise is applied to the sole of the feet^19,30^. It was speculated that this may be a consequence of eliminating sensory cues that would normally help with switching to the appropriate walking pattern, such that the treadmill walking pattern would persevere more without these cues^21–23,32^. We propose instead that these cues may modulate the extent of generalization because the portion of recalibration that generalizes from the treadmill to overground walking may not consist of a single, discrete forward model of the body, but may consist of multiple forward models associated with different sensory cues^15,16,29^. Each model contributes to the control of walking—and can therefore be adapted on the treadmill and/or expressed overground—when the corresponding sensory cue is present in the environment^63–65^. More similar sensory cues between treadmill and overground contexts would result in more forward models being shared across the two contexts, and hence greater generalization.

The same framework may help explain why overground aftereffects are not modulated by walking speed, but treadmill aftereffects are. Consistent with work from Hamzey et al.^17^, we found that overground walking speed does not affect motor generalization. Furthermore, we show that perceptual generalization is also not affected by this manipulation. The robustness of overground aftereffects to walking speed manipulations is in contrast to treadmill aftereffects, which are instead smaller when the walking speed is different than the “slow” speed experienced in adaptation^17,47^. An interesting idea is that the portion of recalibration that does not generalize to overground walking may also consist of multiple forward models, associated with sensory cues such as belt speed, explaining why there is limited generalization of treadmill aftereffects across belt speeds. Because these forward models are specific to the treadmill, they would not contribute to the control of walking overground, potentially explaining why overground aftereffects are not modulated by walking speed. Consistent with this idea, work in the upper extremities has shown that generalization to untrained reaching directions can transfer to the untrained hand^66^.

However, this interpretation is speculative and there are alternative candidate frameworks regarding the organization of forward models. For example, studies exploring the relationship between feedforward and feedback control provide evidence that internal models may be reused and optimized to be shared for different functions^67,68^. Future work is needed to test the relationship between overground transfer and generalization to different treadmill speeds.

It is possible that our findings could be used to inform customizable gait rehabilitation approaches that aim to induce asymmetric changes in joint-level kinematics. As shown in our Supplementary Information, we found significant aftereffects in the joint angles of both limbs during overground generalization. Kambic et al. recently detailed the changes in knee and hip joint motions that occur with split-belt adaptation^69^, and here we showed that these joint-level changes also generalize from treadmill adaptation to overground walking (discussed more in depth in Supplementary Note 1, “Discussion: adaptation alters knee and hip angles in overground walking”). In particular, these results may be useful in designing personalized gait training approaches for clinical populations with intact areas of the brain that are known to be involved in adaptation-based motor learning^29,70–73^ including the cerebellum and its role in connecting with spinal circuits like central pattern generators^74–79^ (e.g., cerebral stroke, cerebral palsy). Beyond the current study, it will be important to continue to improve our understanding of how adaptation-based treadmill training generalizes to overground walking in these clinical populations.

Studies like ours inevitably have some limitations. Our motor analysis focused primarily on step length asymmetry, with the additional analysis of joint kinematics and support times for Experiment 1A. However, walking adaptation is known to change several other measures that were not analyzed here—e.g., forces^80^, joint moments^81^, stability^82^, mechanical and metabolic power^83^, and muscle activity^84^. Therefore, the generalization pattern of these measures, and the underlying mechanisms, may differ from those presented in our study. Additionally, the findings of our study may not be applicable to motor tasks different from split-belt walking adaptation. In particular, it is likely that the mechanisms for generalization described in this study differ from those involved in movement types that are less automatic than walking. This is because the neural control of walking is thought to differ from that of other, less automatic lower limb movements, like tasks involving obstacles, staircase stepping, stepping sideways, etc.^85–87^. Future work is needed to evaluate the pattern and mechanisms of generalization across different tasks and movement types.

In summary, training with devices like split-belt treadmills can produce new walking patterns that are partially linked to the device. This means that some of the adapted pattern will only be expressed on the treadmill, thereby reducing generalization to overground walking. It is known that removal of environmental cues during split-belt treadmill walking can increase overground generalization, but there is room for improvement. Our study specifically demonstrates that recalibration of forward models acquired on the treadmill exhibits partial generalization to overground walking. It also demonstrates that there exists a predefined portion of this recalibration and of the adapted walking pattern that generalizes across contexts, supporting the credit assignment hypothesis for explaining partial generalization. As such, our findings suggest that development of new methods to increase credit assignment to the trainee may be important for boosting the effects of device-based training.

## Methods

### Participants

Fifty neurotypical adults took part in the study (mean ± SD age: 25.0 ± 4.3 years, 34 female and 16 male; Supplementary Table 5 contains demographic information of the participants). All participants were screened for neurologic or motor impairments, were naïve to split-belt treadmill walking, and took part in only one experiment. We complied with all relevant ethical regulations for studies with human participants. The Johns Hopkins Institutional Review Board approved the study protocol (IRB00097573). Written informed consent was obtained from all human participants before participation.

We selected the sample size per group through a power analysis conducted on published data, using the G-Power software^88^ with significance level *α* = 0.05. We evaluated a measure from Leech et al.^44^ that is similar to “perceptual treadmill decay”, albeit after a block of treadmill rather than overground washout. The analysis suggested that a sample size of 9 participants would provide 80% power to detect a difference in the group mean for “perceptual treadmill decay” from 0% or 100%. We selected a sample size of 10 participants to be consistent with several prior studies on generalization after split-belt adaptation^14,17,19,20,30^. We performed an additional power analysis on data from Torres-Oviedo and Bastian^16^ evaluating the same “motor treadmill decay” and “motor overground transfer” measures as our study. This analysis confirmed that a sample size of 10 participants is expected to yield above 80% power for detecting a difference between either measure and 0% or 100%.

### Experimental setup

For the treadmill portions of the study, participants walked on a Motek (Amsterdam, NL) split-belt treadmill with one leg on each treadmill belt. The speeds of the two treadmill belts were controlled separately using a custom program in the D-Flow environment (Motek, Amsterdam, NL). Participants wore a safety harness that did not provide body weight support. For the overground portions of the study, participants walked on a six-meter-long walkway. The experiment was carried out in the dark to limit visual information of the treadmill and overground contexts, as this is known to affect overground transfer^15^. To ensure that transitions between treadmill and overground would not cause washout, participants were instructed to walk off the treadmill backwards (as adaptation does not transfer between backward and forward walking^89^), and they were then transported in a wheelchair to the overground walkway.

### Data collection

For the treadmill portions of the experiment, as well as for the overground portions of the Short Preferred and Long Preferred groups in Experiment 1 and the Reference group in Experiment 2, kinematic data was recorded at 100 Hz using a Vicon motion capture system (Oxford, UK). Reflective markers were placed bilaterally over the second and fifth toes (metatarsal heads), heel (calcaneus), medial and lateral ankle (medial and lateral malleoli), shank, medial and lateral knee (medial and lateral femoral epicondyles), thigh, hip (greater trochanter), anterior, posterior and lateral pelvic bone (anterior and posterior superior iliac spines, and iliac crest), as well as on the torso over the jugular notch, xiphoid process, 7th cervical vertebrae, and 10th thoracic vertebrae. For the overground portions of the Short Slow group in Experiment 1 and the Switch group in Experiment 2, participants walked on a Zeno Walkway (ProtoKinetics, Havertown, PA), which recorded spatial and temporal information on foot contact using force sensors.

### Experimental protocol

Experimental paradigms for Experiments 1 and 2 are shown in Figs. 1a and 5a. Each “overground pass” was six meters long. Participants first walked overground for 20 passes. All participants then walked on the treadmill with both belts tied at 0.5 m/s (baseline phase). The baseline phase consisted of four blocks of 1 min each; after each of the first three blocks, participants performed one iteration of the speed match task, which is described below. Participants then walked in a 3:1 split condition (right speed at 1.5 m/s and left speed at 0.5 m/s) for 20 min (adaptation phase), with a 10-second tied-belt catch trial after 15 min (both belts at 0.5 m/s). Immediately after adaptation, participants performed an iteration of the speed match task. They then transitioned to walking overground; participants in the Long Preferred group of Experiment 1 walked overground for 80 passes, while all other participants walked overground for 30 passes. The number of passes in overground baseline and post-adaptation (for all groups except Long Preferred) were chosen to match previous work^15^—which showed this number of passes provides reliable motor measures and can effectively wash out overground aftereffects post-adaptation. The overground walking speed in both baseline and post-adaptation blocks differed between the groups, as described below.

The remainder of the experimental protocol after post-adaptation overground walking differed for Experiments 1 and 2. Participants in Experiment 1 returned to walking on the treadmill for a total of 16.5 min, with alternating blocks of tied-belt walking (both belts at 0.5 m/s) and speed match tasks (Fig. 1a). Participants in Experiment 2 returned to the treadmill for an “extended” speed match task block: they first performed a speed match task (see section “Perceptual tests: speed match task and questionnaire”), and then continued to walk for an additional 90 seconds on belt speeds that they perceived as being equal. We chose this set up as we believed it would minimize sensory prediction error from the belt speeds, and hence reduce either washout or re-adaptation. After the extended speed match task, the Switch group returned to walking overground for 20 passes, and we continued to alternate between extended speed match task blocks on the treadmill and overground walking blocks of 20 passes for a total of 4 overground blocks and 4 extended speed match tasks (Fig. 5a, top). The reference group performed the first overground walking block and all treadmill extended speed match tasks but did not walk overground for post-adaptation blocks 2–4. Instead, between extended speed match tasks, participants in this group walked off the treadmill and sat in the wheelchair for 6 min and 45 s (which was found to be the average time of post-adaptation overground blocks 2–4 for the Switch group). We selected the number of passes in post-adaptation overground blocks 2–4 to be shorter than post-adaptation overground block 1 to limit the overall duration of the experiment and hence the effect of fatigue. We expected that, even if participants had aftereffects in these blocks, these would be smaller and washout faster than in the first block. Furthermore, previous work has shown that 20 passes of 6 meters are typically enough to washout the aftereffects^16^.

### Overground walking speed

Participants in the Short and Long Preferred groups of Experiment 1 walked at their preferred walking speed during baseline and post-adaptation overground blocks; that is, no instruction regarding the walking speed was given. Participants in all other groups were instructed to walk at a speed of ~0.5 m/s (i.e., the “slow” speed experienced on the treadmill) in all overground blocks. For groups walking on the Zeno electronic walkway (ProtoKinetics, Havertown, PA), the average walking speed for each overground pass was automatically measured by the walkway software; we used a timer to measure walking speed for each pass of the other groups. Participants were given feedback on their walking speed after each pass throughout the experiment. A speed was considered acceptable if it was between 0.45 and 0.55 m/s, otherwise participants were told they moved “too slow” or “too fast”. We included all trials regardless of success because we could not recollect unsuccessful trials in post-adaptation due to washout/unlearning. Hence, before the start of the experiment, participants underwent an overground practice block to ensure that they could walk at the desired speed overground; the experiment started after they could successfully walk between 0.45 and 0.55 m/s for 8 of 10 consecutive passes (no practice was given to the “Preferred” groups).

### Perceptual tests: speed match task and questionnaire

Our primary perceptual test was the “speed match task”, which measures perception of leg speed. The task is inspired by that designed by Vazquez et al.^42^, and based on the psychophysical method of adjustment^90^. The task setup is depicted in Fig. 2b. The left belt moved at a constant speed of 0.5 m/s. The right belt started at a speed of 0.3 m/s and was then controlled by the participants, who were instructed to “make the right belt move as fast as the left belt” by steering an Xbox controller’s joystick (FiveStar USB controller for Xbox 360, Microsoft). When the right speed was in the range of 0.475 to 0.525 m/s (that is, less than 25 mm/s different than the left speed), steering the stick changed the right speed by 10 mm/s; otherwise, the change was 35 mm/s. Each iteration of the task lasted 30 seconds and a time bar was projected on a screen in front of the treadmill. To limit visual or auditory information of the treadmill belts, the room lights were kept off and white noise was played through speakers during the task.

Participants in Experiment 1 also completed a questionnaire after the post-adaptation overground walking block, where they were instructed to circle either “*as expected”* or *“not as expected”* in response to the following two prompts:

1. at the beginning of this trial, the right leg felt it was moving: *as expected/not as expected.*
2. at the end of this trial, the right leg felt it was moving: *as expected/not as expected.*

### Data analysis: step length asymmetry

We chose step length asymmetry as the kinematic parameter of interest, as it has been shows to adapt robustly on the split-belt treadmill^91^ and transfer to overground walking^15^. For each stride—defined as the period from a heel strike to the following heel strike of the same leg—we computed:

1 $${\rm{step}}\,{\rm{length}}\,{\rm{asymmetry}}=\frac{({{\rm{SL}}}_{{\rm{R}}}-{{\rm{SL}}}_{{\rm{L}}})}{({{\rm{SL}}}_{{\rm{R}}}+{{\rm{SL}}}_{{\rm{L}}})}$$

Where step lengths SL_R_ and SL_L_ are computed as the anterior-posterior distance between the feet at right and left heel strikes respectively (positive values indicate that the heel strike foot is ahead of the other). The distance between the feet was computed using the lateral ankle markers for the data recorded with Vicon, and the posterior bounds of the feet for data recorded with the Zeno Walkway. A step length asymmetry of zero indicates symmetric steps.

We then computed variables to characterize the participants’ motor behavior at different stages of the experiment. Each variable was computed by averaging the step length asymmetry parameter over strides taken at a specific time epoch in the experiment, as defined below. For both experiments, we computed:

- “overground baseline” = average of all strides in the overground baseline block.
- “treadmill baseline” = average of all strides in the treadmill baseline blocks.
- “catch trial” = average of the first 5 strides in the catch trial (10-second tied-belt block during treadmill adaptation).
- “initial overground post-adaptation” = average of the first 5 strides in the (first) overground post-adaptation block.

For Experiment 1, we computed:

- “initial treadmill post-adaptation” = average of the first 5 strides in the first tied-belt block in treadmill post-adaptation.

For the Switch group in Experiment 2, we computed:

- “start of post-adaptation overground 2” = average of the first 5 strides in post-adaptation overground 2.
- “start of post-adaptation overground 3” = average of the first 5 strides in post-adaptation overground 3.
- “start of post-adaptation overground 4” = average of the first 5 strides in post-adaptation overground 4.
- “end of post-adaptation overground 1” = average of the last 5 strides in post-adaptation overground 1.
- “end of post-adaptation overground 2” = average of the last 5 strides in post-adaptation overground 2.
- “end of post-adaptation overground 3” = average of the last 5 strides in post-adaptation overground 3.

Note that all of these measures have the normalized units of step length asymmetry.

We used these variables to compute measures of motor generalization, similar to previous work^15,16^. For both experiments, we computed:

2 $$\begin{array}{l}{\rm{motor}}\,{\rm{overground}}\,{\rm{transfer}}\,( \% )\\=\frac{{\rm{initial}}\,{\rm{overground}}\,{\rm{post}}\mbox{-}{\rm{adaptation}}\ -\ {\rm{overground}}\,{\rm{baseline}}}{{\rm{catch}}\,{\rm{trial}}\ -\ {\rm{treadmill}}\,{\rm{baseline}}}\,*\, 100\end{array}$$

For Experiment 1, we computed:

3 $$\begin{array}{l}{\rm{motor}}\,{\rm{treadmill}}\,{\rm{decay}}\,( \% )\\=\frac{{\rm{catch}}\,{\rm{trial}}\ -\ {\rm{initial}}\,{\rm{treadmill}}\,{\rm{post}}\mbox{-}{\rm{adaptation}}}{{\rm{catch}}\,{\rm{trial}}\ -\ {\rm{treadmill}}\,{\rm{baseline}}}\ast 100\end{array}$$

For the Switch group in Experiment 2, we computed the following measure for blocks “post-adaptation overground 2”, “3” and “4” (*N* and *N-1* refer to the block number):

4 $$\begin{array}{ll}{\rm{additional}}\,{\rm{motor}}\,{\rm{overground}}\,{\rm{transfer}}_{\,{\rm{overground}}\,{{N}} }\,( \% )\\=\displaystyle{\frac{{{\rm{start}}}{\,{\rm{of}}} {\,{\rm{post}}\mbox{-}{\rm{adaptation}}\,{{\rm{overground}}}\,{{N}}}\ -\ {{\rm{end}}}{\,{\rm{of}}} {\,{\rm{post}}\mbox{-}{\rm{adaptation}}\,{{\rm{overground}}}\,{{N}}\mbox{-}{{1}}}}{ {\rm{catch}}\,{\rm{trial}}\ -\ {\rm{treadmill}}\,{\rm{baseline}}}\,*\, 100}\end{array}$$

### Data analysis: walking speed and number of strides

For Experiment 1B, we additionally evaluated overground walking speed and overground number of strides for all groups.

For each overground pass of 6 meters, we computed walking speed as the distance traveled over time of the pass (this may differ from that used during the experiment to provide feedback to the participants in the Short Slow group). Both distance and time were computed using the interval from the start of the first stride to the end of the last stride used for the step length asymmetry analysis. Similar to the step length asymmetry analysis, the distance was computed using the lateral ankle markers for the data recorded with Vicon, and the posterior bounds of the feet for data recorded with the Zeno Walkway.

For the Short Slow group, we used walking speed data from individual passes to perform a within-participant analysis comparing walking speed to the 0.45 m/s–0.55 m/s target range—described below in “Statistical analysis”.

For within-group and between-group analyses for all groups, we pre-averaged the walking speed within-participant over all passes taken within an experimental block, to obtain the walking speed in overground baseline and overground post-adaptation. Similarly, for each participant we computed the total number of strides taken in each block. Hence, for each participant we obtained the measures:

- “walking speed in overground baseline” = average walking speed over all passes in the overground baseline block.
- “walking speed in overground post-adaptation” = average walking speed over all passes in the overground post-adaptation block.
- “number of strides in overground baseline” = total number of strides taken across all passes in the overground baseline block.
- “number of strides in overground post-adaptation” = total number of strides taken across all passes in the overground post-adaptation block.

### Data analysis: perceptual bias

For the speed match tasks, the perceptual measure of interest was bias in the perception of leg speed, as this measure has been shown to recalibrate robustly with split-belt walking^42,50^.

For each iteration of the speed match task, we computed:

5 $${\rm{perceptual}}\,{\rm{bias}}={\rm{final}}\,{\rm{right}}\,{\rm{belt}}\,{\rm{speed}}-{\rm{left}}\,{\rm{belt}}\,{\rm{speed}}$$

Where the left belt speed is fixed at 0.5 m/s. The perceptual bias represents the belt speed difference reported to feel as “equal speeds” by the participants.

In particular, we used this variable evaluated at the following time points to assess generalization:

- bias _baseline_: perceptual bias averaged across the three baseline speed match tasks.
- bias _adaptation_: perceptual bias captured by the speed match task at the end of treadmill adaptation.
- bias _post-adaptation__1_: perceptual bias captured by the first speed match task in treadmill post-adaptation.

We then computed a measure of perceptual generalization–perceptual treadmill decay:

6 $${\rm{perceptual}}\,{\rm{treadmill}}\,{\rm{decay}}\,( \% )=\frac{{{\rm{bias}}}_{\,{\rm{adaptation}}}\ -\ {{\rm{bias}}}_{\,{\rm{post}\mbox{-}{adaptation}}\,{1}}}{{{\rm{bias}}}_{\,{\rm{adaptation}}}\ -\ {{\rm{bias}}}_{\,{\rm{baseline}}}}\,*\, 100$$

In Experiment 2, we additionally computed the following measure for each pair of consecutive treadmill blocks in post-adaptation (“treadmill 2-1”, “3-2”, and “4-3”; *N* and *N-1* refer to the block number):

7 $$\begin{array}{ll}{\rm{additional}}\,{\rm{motor}}\,{\rm{treadmill}}\,{\rm{decay}}_{\,{\rm{treadmill}}\,{{N}}-{{N}}\mbox{-}{{1}}}\,( \% )\\=\displaystyle{\frac{{{\rm{bias}}}_{\,{\rm{post}}\mbox{-}{\rm{adaptation}}\,{{N}}}\ -\ {{\rm{bias}}}_{\,{\rm{post}}\mbox{-}{\rm{adaptation}}\,{{N}}\mbox{-}{{1}}}}{{{\rm{bias}}}_{\,{\rm{adaptation}}}\ -\ {{\rm{bias}}}_{\,{\rm{baseline}}}}\,* \,100}\end{array}$$

### Statistical analysis

All statistical tests were performed in MATLAB with significance level *α* = 0.05 (two-sided), using a bootstrap procedure (all tests are summarized in Tables 1 and 2). To assess between-group differences in our measures of interest, we computed bootstrap confidence intervals (CI) for the difference of the means between the two groups. The analysis for each comparison consisted of the following steps:

1. We generated 10,000 bootstrapped samples, each comprising 20 participants, with 10 participants resampled with replacement from each group independently.
2. For each bootstrapped sample, denoted as *b*, we computed a bootstrap replication of the difference of the means between the two groups for the measure of interest: $\varDelta \mu (b)={\mu }_{{\rm{group}}1}(b)-{\mu }_{{\rm{group}}2}(b)$. Here, *μ*_group1_ (*b*) represents the measure of interest averaged over the 10 participants resampled from group 1, and *μ*_group2_ (*b*) is analogous but from group 2.
3. We computed the 95% CI for the difference of means, corresponding to significance level *α* = 0.05.
4. For each family of related tests, correction for multiple comparisons was performed using the False Discovery Rate (FDR) method^92^. Specifically, the significance level was adjusted to ${a}_{{\rm{corr}}}={a}^{\ast }\frac{{{R}}}{{{m}}}={0.05}^{\ast }\frac{{{R}}}{{{m}}}$, where *m* represents the number of tests evaluated in the family, and *R* represents the number of tests deemed significant (i.e., indicating significant difference between groups). Families of related tests are defined in Tables 1 and 2.

**Table 1:** Description of statistical tests for Experiment 1

Measure of interest	Null hypothesis	Family of tests	Test group(s)
Within-group statistical analysis			
Motor overground transfer	Mean of test group = 0%	*m* = 3	Short Preferred, Long Preferred, Short Slow
Motor treadmill decay	Mean of test group = 0%		
Perceptual treadmill decay	Mean of test group = 0%		
Motor overground transfer	Mean of test group = 100%	*m* = 3	Short Preferred, Long Preferred, Short Slow
Motor treadmill decay	Mean of test group = 100%		
Perceptual treadmill decay	Mean of test group = 100%		
Step length asymmetry in overground baseline	Mean of test group = 0	*m* = 3	Short Preferred
Step length asymmetry in treadmill baseline	Mean of test group = 0		
Step length asymmetry in catch trial	Mean of test group = 0		
Perceptual bias in baseline	Mean of test group = 0	*m* = 2	Short Preferred
Perceptual bias in adaptation	Mean of test group = 0		
Walking speed in overground baseline	Mean of test group = 0.5 m/s	*m* = 2	Short Slow
Walking speed in overground post-adaptation	Mean of test group = 0.5 m/s		
Between-group statistical analysis			
Motor overground transfer	Mean of test group = mean of Short Preferred	*m* = 3	Long Preferred, Short Slow
Motor treadmill decay	Mean of test group = mean of Short Preferred		
Perceptual treadmill decay	Mean of test group = mean of Short Preferred		
Walking speed in overground baseline	Mean of test group = mean of Short Preferred	*m* = 2	Long Preferred, Short Slow
Walking speed in overground post-adaptation	Mean of test group = mean of Short Preferred		
Number of strides in overground baseline	Mean of test group = mean of Short Preferred	*m* = 2	Long Preferred, Short Slow
Number of strides in overground post-adaptation	Mean of test group = mean of Short Preferred		

*“Measure of interest”* measures used for the analysis, *“Null Hypothesis”* comparison evaluated in this test, *“Family of tests”* related tests that are considered together for the correction for multiple comparisons, and corresponding “*m*”, *“Test group(s)”* groups for which this analysis is carried out.

**Table 2:** Description of statistical tests for Experiment 2

Measure of interest	Null hypothesis	Family of tests	Test group(s)
Within-group statistical analysis for Experiment 2			
Motor overground transfer	Mean of test group = 0%	*m* = 2	Switch, Reference
Perceptual treadmill decay	Mean of test group = 0%		
Motor overground transfer	Mean of test group = 100%	*m* = 2	Switch, Reference
Perceptual treadmill decay	Mean of test group = 100%		
Additional motor overground transfer, for overground 2	Mean of test group = 0%	*m* = 3	Switch
Additional motor overground transfer, for overground 3	Mean of test group = 0%		
Additional motor overground transfer, for overground 4	Mean of test group = 0%		
Additional perceptual treadmill decay, for treadmill 2-1	Mean of test group = 0%	*m* = 3	Switch, Reference
Additional perceptual treadmill decay, for treadmill 3-2	Mean of test group = 0%		
Additional perceptual treadmill decay, for treadmill 4-3	Mean of test group = 0%		
Between-group statistical analysis for Experiment 2			
Motor overground transfer	Mean of test group = mean of Reference group	*m* = 2	Switch
Perceptual treadmill decay	Mean of test group = mean of Reference group		
Step length asymmetry in overground baseline	Mean of test group = mean of Reference group	*m* = 3	Switch
Step length asymmetry in treadmill baseline	Mean of test group = mean of Reference group		
Step length asymmetry in catch trial	Mean of test group = mean of Reference group		
Perceptual bias in baseline	Mean of test group = mean of Reference group	*m* = 2	Switch
Perceptual bias in adaptation	Mean of test group = mean of Reference group		
Additional perceptual treadmill decay, for treadmill 2-1	Mean of test group = mean of Reference group	*m* = 3	Switch
Additional perceptual treadmill decay, for treadmill 3-2	Mean of test group = mean of Reference group		
Additional perceptual treadmill decay, for treadmill 4-3	Mean of test group = mean of Reference group		

*“Measure of interest”* measures used for the analysis, *“Null Hypothesis”* comparison evaluated in this test, *“Family of tests”* related tests that are considered together for the correction for multiple comparisons, and corresponding “*m*”, *“Test group(s)”* groups for which this analysis is carried out.

For each computed CI, we interpreted a measure of interest to be significantly different between two groups if the CI did not overlap zero.

We used a similar procedure for within-group statistical analyses. To compare each measure of interest against a specific value (e.g., 0%), we computed bootstrap confidence intervals (CI) for the mean for each measure. First, we generated 10,000 bootstrapped samples of 10 participants resampled with replacement from the group. For each bootstrapped sample, we computed a bootstrap replication of the group mean for the measure of interest by averaging over participants in that sample. We then computed the confidence interval corrected for multiple comparisons^92^ as explained in steps (3) and (4) above. Each test was considered significant if the CI did not overlap the value specified in the null hypothesis^93–95^—see Tables 1 and 2.

For each group in Experiment 1B, we used an analogous procedure for the within-participant analysis of overground walking speed. To assess walking speed in overground post-adaptation—which consisted of 30 passes of 6-meters—for each participant we generated 10,000 bootstrap samples of 30 passes resampled with replacement from the overground post-adaptation block. Similarly, to assess walking speed in overground baseline—which consisted of 20 passes—for each participant we generated 10,000 bootstrap samples of 20 passes resampled with replacement from the overground baseline block. For each bootstrap sample, we then computed a replication of the mean walking speed in each block. We used this to compute the confidence interval for the mean walking speed in overground baseline and overground post-adaptation for each participant. We then corrected for multiple comparisons as explained in steps (3) and (4) above. We define a family of tests to be an individual participant’s confidence intervals for overground baseline and overground post-adaptation, so that *m* = 2. Each test was considered significant if the CI did not overlap the range of 0.45–0.55 m/s.

### Inclusion and ethics

We have given thoughtful consideration to research contributions and authorship as part of efforts to promote equitable authorship within research collaborations.

### Reporting summary

Further information on research design is available in the Nature Research Reporting Summary linked to this article.

### Supplementary information

Supplementary Information

Reporting Summary

## Data Availability

The datasets generated and analyzed during the current study are available in the Dryad repository: Rossi et al.^96^.

## Code availability

The code used for the analyses is available in the Dryad repository: Rossi et al.^96^.
