# Supplementary material for: Understanding mechanisms of generalization following locomotor adaptation
Source: NPJ Sci Learn. 2024 Jul 23;9:48. doi: 10.1038/s41539-024-00258-2 (PMC11266392; doi:10.1038/s41539-024-00258-2)
Supplement: Supplementary file 1 — Supplementary Information [file 41539_2024_258_MOESM1_ESM.pdf]

Supplementary information for:

Understanding mechanisms of generalization following locomotor adaptation

Cristina Rossi[a,b], Ryan T. Roemmich[b,c], Amy J. Bastian[a,b,*]

[a] Department of Neuroscience, The Johns Hopkins University School of Medicine, Baltimore, Maryland, 21205, USA; [b] Center for Movement Studies, Kennedy Krieger Institute, Baltimore, Maryland, 21205, USA; [c] Department of Physical Medicine and Rehabilitation, The Johns Hopkins University School of Medicine, Baltimore, Maryland, 21205, USA. *Email: bastian@kennedykrieger.org.

Content

Supplementary Note 1: Analysis of different gait kinematic parameters **2**

Results: overground aftereffects in joint angles, segment advancements, and support times 2

Discussion: adaptation alters knee and hip angles in overground walking 6

Methods 7

Supplementary Note 2: Control analyses for Experiment 2 ....**9**

Supplementary Tables **10**

Supplementary Table 1. Overground aftereffects in the advancement of leg segments and joint angles at heel strike and toe off events 10

Supplementary Table 2. Overground aftereffects in support times.. 11

Supplementary Table 3. Overground aftereffects in peak flexion and extension of the hip and knee.... 12

Supplementary Table 4. Overground walking speed of individual participants in all groups of Experiment 1 13

Supplementary Table 5. Demographics of the participants 14

Supplementary References **15**

Supplementary Figures **16**

Supplementary Figure 1. Individual participant data for Experiment 1A 17

Supplementary Figure 2. Additional results for Experiment 1A. 18

Supplementary Figure 3. Overground aftereffects in joint kinematic patterns, segment configuration and temporal parameters. 19

Supplementary Figure 4. Overground aftereffects in joint kinematic patterns and segment configuration, with legs overlayed to highlight asymmetries 20

Supplementary Figure 5. Motor and perceptual timeseries results for Long Preferred and Short Slow groups of Experiment 1B 21

Supplementary Figure 6. Results to confirm the manipulations of Experiment 1B................................ 22

Supplementary Figure 7. Overground walking speed for individual participants in Experiment 1 23

Supplementary Figure 8. Additional results for Experiment 2.. 24

Supplementary Figure 9. Individual participant data for Experiment 2 25

Supplementary Note 1:

Analysis of different gait kinematic parameters

Results: overground aftereffects in joint angles, segment advancements, and support times

In the main text, we focused on the generalization of treadmill aftereffects in step length asymmetry to overground walking. Yet, split-belt adaptation has been shown to change several different aspects of walking, at least on the treadmill[1,2]. Therefore, we here look to see how kinematics other than step length asymmetry are altered during overground walking following split-belt adaptation.

We found changes in both spatial and temporal gait parameters, demonstrating that treadmill adaptation can alter stance time, knee flexion, and hip extension in overground walking. Notably, we observed changes in interlimb coordination, suggesting that our treadmill training approach can effectively alter spatial and temporal gait asymmetries. Although our analysis was not tailored to a specific neurological condition, our results suggest potential benefits for post-stroke walking rehabilitation, addressing issues like stiff knee gait, impaired paretic propulsion, and asymmetrical leg movements.

We described changes in specific spatiotemporal parameters below. We compared joint angles, advancement of leg segments, and support times, between overground baseline and the start of the post-adaptation overground walking block (Supplementary Figure 3-4). We will refer to the legs as “fast” or “slow” (instead of “right” or “left”) because the direction of the aftereffects depends on which leg was placed on the fast belt in adaptation. The analyses are performed on data from the Short Preferred group of Experiment 1.

Slow step is shortened by more flexed slow knee and less extended fast hip at slow heel strike. We first evaluated changes in joint angles at heel strike of each leg, and their effect on step length. Supplementary Figure 3a shows the intralimb coordination across hip and knee angles in baseline (dashed black line) and post-adaptation overground walking (solid blue/red lines for the slow/fast leg); the timing of gait events is indicated by tick marks perpendicular to the angle trace. Supplementary Figure 3b shows the configuration of limb segments at each gait event during overground baseline (dashed black lines) and post-adaptation (solid color lines), with segment advancement projection underneath (dashed and shaded for baseline, solid for post-

adaptation). Segment advancements at slow heel strike (shs) and fast heel strike (fhs) constitute the contribution of each segment to the slow and fast step lengths respectively. We observed that limb configuration seemed different between baseline and post-adaptation at slow heel strike, but not at fast heel strike. Indeed, we found that the slow step length was significantly shorter post-adaptation as compared to baseline (post-adaptation minus baseline difference in slow step length = -0.1258 [-0.1719, -0.0871] m, mean [CI]), while the fast step length did not change (difference = -0.0056 [-0.0525, 0.0342] m).

The shortening of the slow step length was achieved by concurrent changes in the slow and fast legs at slow heel strike. Specifically, the slow knee was significantly more flexed post-adaptation than in baseline at slow heel strike (difference = 8.8771 [5.1571, 13.9720] deg; Supplementary Figure 3a left, and dark blue in Supplementary Figure 3c “shs”), which resulted in less advancement from the slow shank (difference = -0.0416 [-0.0585, -0.0237] m; dark blue in Supplementary Figure 3b-c “shs”). Additionally, the fast hip was less extended at post-adaptation slow heel strike (difference = 5.3979 [3.8990, 7.1091] deg; Supplementary Figure 3a middle, and orange in Supplementary Figure 3c “shs”), which reduced the advancement from both the fast thigh and shank (difference for thigh = -0.0385 [-0.0498, -0.0278] m, shank = -0.0403 [-0.0524, -0.0276] m; orange and red in Supplementary Figure 3b-c “shs”). There was no change between baseline and post-adaptation slow hip angle, slow thigh advancement, fast knee angle, and pelvis advancement at slow heel strike (all CI_{LB} < -0.007m or -1.8deg and all CI_{UB} > 0.0007m or 1deg, all statistics reported in Supplementary Table 1).

Changes in configuration of both the slow and fast leg persisted at fast toe off, such that the distance between the ankles was also shorter during post-adaptation fast toe off as compared to baseline (difference = -0.1326 [-0.1791, -0.0934] m). Similar to slow heel strike, the slow knee was more flexed (difference = 2.4474 [1.3257, 3.4564] deg; Supplementary Figure 3a left, and dark blue in Supplementary Figure 3c “fto”) and the slow shank was in a less advanced position at fast toe off (difference = -0.0494 [-0.0601, -0.0379] m; dark blue in Supplementary Figure 3b-c “fto”). The slow leg advancement was further reduced by changes in slow hip angle, which was less flexed at fast toe off in post-adaptation than in baseline (difference = -5.3072 [-6.5908, -4.1733] deg; Supplementary Figure 3a left, and light blue in Supplementary Figure 3c “fto”), such that the slow thigh was in a less advanced position (difference = -0.0318 [-0.0409, -0.0229] m; light blue in Supplementary Figure 3b-c “fto”). The fast leg advancement was reduced at post-adaptation fast toe off in a manner consistent with slow heel strike: the fast hip was less extended (difference = 4.5459 [2.8987, 6.7686] deg; Supplementary Figure 3a middle, and orange in

Supplementary Figure 3c “fto”), such that the fast thigh and fast shank advanced less (difference for thigh = -0.0307 [-0.0469, -0.0184] m, shank = -0.0152 [-0.0243, -0.0077] m; red and orange in Supplementary Figure 3b-c “fto”). Note that the fast knee was also more flexed (difference = 2.2048 [1.3118, 3.2163] deg; Supplementary Figure 3a middle, and red in Supplementary Figure 3c “fto”); this would contribute to lengthening the advancement of the fast shank but the effect was masked by the less extended fast hip.

We found no change between baseline and post-adaptation in the distance between the ankles at slow toe off (difference = -0.0041 [-0.0511, 0.0363] m), or in any of the joint angles and segment advancements at fast heel strike and slow toe off (all CI_{LB} < -0.002m or -0.1deg and all CI_{UB} > 0.0005m or 0.1deg, all statistics reported in Supplementary Table 1).

Longer fast double support time, and reduced peak extension of slow knee and fast hip, contribute to the altered limb configuration at slow heel strike. Two factors may contribute to the observed aftereffects in limb configuration at slow heel strike and fast toe off: 1) a change in the timing of gait events, or 2) a change in knee-hip joint coordination. To assess the former, we evaluated aftereffects in support times, which are depicted as insets in Supplementary Figure 3b. We found that the fast double support time – i.e., the time between slow heel strike and fast toe off – was significantly longer post-adaptation (difference = 0.0917 [0.0742, 0.1106] s; Supplementary Figure 3b inset “DS_f”) as compared to baseline. There was no significant aftereffect in slow single support, slow double support, or fast single support times (all CI_{LB} < -0.01s and all CI_{UB} > 0.003s, all statistics reported in Supplementary Table 2).

We assessed changes in joint coordination by evaluating peak flexion and extension of the hip and knee. When evaluating the slow leg (Supplementary Figure 3a, left), we found that the peak extension of the slow knee was significantly reduced post-adaptation as compared to baseline (difference = -5.2376 [-7.1574, -3.2710] deg; Supplementary Figure 3a left inset). This reflect the less extended knee angle at slow heel strike in post-adaptation discussed above (blue versus black circles in Supplementary Figure 3a left). The slow knee-hip angle trace was otherwise similar between baseline and post-adaptation (Supplementary Figure 3a left, dashed black and solid blue lines are similar), and there were no significant aftereffects in peak knee flexion, peak hip flexion or peak hip extension of the slow leg (all CI_{LB} < -1deg and all CI_{UB} > 0.6deg, all statistics reported in Supplementary Table 3; Supplementary Figure 3a left inset). These results, together with the aftereffect in fast double support time discussed above, suggest that the slow leg aftereffects at fast toe off is primarily due to the change in the timing of this event. Post-adaptation,

fast toe-off occurs later in the gait cycle (compare solid blue to dashed black "fto" tick marks), resulting in the knee being more flexed and the hip more extended, mirroring the natural transition of joint angles during the stance phase.

When evaluating the fast leg (Supplementary Figure 3a, right), we noted a distinct difference in the fast knee-hip angle trace between baseline and post-adaptation, that persisted for a larger portion of the stride cycle (red trace seems overall “smaller” than the dashed black trace). In particular, both fast knee and hip joints experienced smaller ranges of angles post-adaptation compared to baseline: we found a significant reduction in peak knee flexion (difference = -2.7164 [-5.7152, -0.3951] deg), peak knee extension (difference = -1.2569 [-2.7257, -0.0765] deg), and peak hip extension (difference = -3.4684 [-5.1900, -2.2463] deg), although not in peak hip flexion (difference = -0.2107 [-1.8423, 1.3373] deg; Supplementary Figure 3a right inset). These findings, combined with the absence of significant aftereffects in slow double support and fast single support times as described earlier, suggest that the aftereffects in the fast leg at slow heel strike primarily stem from changes in joint coordination. Specifically, the reduced hip extension observed at slow heel strike is likely linked to the overall decrease in peak hip extension across the gait cycle post-adaptation. In contrast, the aftereffects in the fast leg at fast toe-off may arise from both changes in event timing and joint coordination. The delayed timing of fast toe-off in the post-adaptation gait cycle leads to increased knee flexion and decreased hip extension. Additionally, the overall reduction in peak hip extension throughout the gait cycle also contributes to the decreased hip extension at fast toe-off.

Asymmetric step lengths are due to slow hip extending more than the fast hip when trailing, and slow knee extending less than fast knee when leading. We confirm the contribution of individual leg changes to step length asymmetry aftereffects by displaying the asymmetries between the legs. Specifically, in Supplementary Figure 4, we present overlaid post-adaptation joint angle traces and limb configurations of each leg relative to their own gait cycle. The fast knee appears more extended than the slow knee at heel strike of the same leg. This contributes to the fast leg advancing more than the slow leg, and is consistent with positive step length asymmetry aftereffects (i.e., fast step longer than slow step). Additionally, we observe that the fast hip appears to extend less than the slow hip for a large portion of the gait cycle, including the heel strike of the opposite leg and the subsequent toe-off of the same leg. This contributes to fast leg trailing less behind the body compared to the slow leg, resulting in a shorter slow step, which is again consistent with positive step length asymmetry aftereffects.

In sum, we show that overground aftereffects in step length asymmetry reflect the shortening of the slow step length but not the fast step length. This is achieved by changing the configuration of both limbs at slow heel strike: the slow foot is less ahead of the body because the slow knee extends less, and the fast foot is less behind the body because fast hip extends less. We observed that similar changes persist at fast toe off. We finally showed that aftereffects are achieved by reducing the range of motion of the slow knee, and fast hip and knee, as well as by spending more time in fast double support after slow heel strike.

Discussion: adaptation alters knee and hip angles in overground walking

Work in overground generalization has primarily focused on aftereffects in step length asymmetry, with some studies parsing spatial from temporal contributions to the aftereffect[3–7], and in the asymmetry of support times[8–12]. We here dissected the aftereffect further and investigated the contribution of each joint angle and leg segment advancement to step length asymmetry. We found that after adaptation, both feet were closer to the body midline at slow heel strike, shortening the slow step length: the slow shank led less in front of the body because of the reduced slow knee extension, and both fast thigh and shank trailed less behind the body because of the reduced fast knee extension. This result appears at odds with previous suggestions that overground aftereffects reflect changes to the trailing, but not the leading position of the feet[13,14]. We speculate that this discrepancy may be explained by the smaller adaptation speed ratio in these studies – 2:1 in contrast to the 3:1 ratio used here. Indeed, larger speed ratios have been associated with larger aftereffects[1,2]. Finally, we found that overground aftereffects persisted at fast toe off, but dissipated at fast heel strike such that, in line with previous findings[14], the fast step length was similar between baseline and post-adaptation.

Methods

The additional analyses were performed on data from the Short Preferred group of Experiment 1. We computed gait parameters for each stride, consisting of the following events: shs (slow heel strike), fto (fast toe off), fhs (fast heel strike), sto (slow toe off), shs (subsequent slow heel strike).

We computed sagittal joint angles for each leg. We computed knee angle as the angle formed by greater trochanter, lateral knee, and lateral ankle markers. We computed hip angle as the angle between the vertical and the vector from the greater trochanter to the lateral knee markers. We evaluated joint angles at each event in the stride cycle. We also computed peak flexion and extension as the maximum and minimum angle experienced in a stride. For joint angle traces and measures of joint angles at events in the gait cycle, positive angles denote flexion, while negative angles indicate extension. For peak flexion and peak extension measures, positive angles signify larger peaks (i.e., a larger peak extension implies that the joint is more extended).

We also evaluated the sagittal configuration of the following limb segments: pelvis (segment between right and left trochanter markers), thigh (segment between trochanter and lateral knee) of each leg, and shank (segment between lateral knee and lateral ankle marker) of each leg. For each event in the stride cycle, we computed segment advancement as the projection of each segment on the anterior-posterior axis. For shs and fto, each segment projection was positive if the respective markers were positioned in the following order from anterior to posterior: slow ankle, slow knee, slow trochanter, fast trochanter, fast knee, fast ankle. The sign was opposite for fhs and sto. Note that step length equals the sum of segment advancements at heel strike; similarly, we evaluated overall distance between the ankles at toe offs.

We finally evaluated the timing between events in each stride cycle, defined as follows: fast double support (DS_f) = time from shs to fto, slow single support (SS_s) = time from fto to fhs, slow double support (DS_s) = time from fhs to sto, fast single support (SS_f) = time from sto to subsequent shs).

For each gait parameter of interest, we evaluated the time epochs of overground baseline (average of all strides), and initial overground post-adaptation (average of the first 5 strides immediately after adaptation), and computed the measure of interest:

$$\text{overground aftereffect} = \text{initial overground post-adaptation} - \text{overground baseline}$$

For each measure of interest, we tested the null hypothesis that the mean of the measure of the Short Preferred group equals zero, using the procedure descried in the main text (Methods, Statistical analysis). Significant tests indicate that there is a significant overground aftereffect for the respective gait parameter. We summarize the measures of interest below, including the parameter “m” representing the size of the family of related statistical tests, used for correction for multiple comparisons:

- Overground aftereffect in fast step length, slow step length, slow leg advancement at fto, fast leg advancement at sto, compared to zero (m=4).
- Overground aftereffect in support times, compared to zero (fast/slow single/double support time; m=4).
- Overground aftereffect in joint angles (knee and hip of each leg) and segment advancement (thigh and shank of each leg, and pelvis) at each gait cycle event, compared to zero (m=9, each event – shs, fto, fhs, sto – consisting of a separate comparison group)
- Overground aftereffect in peak flexion and extension of joint angles (knee and hip of each leg), compared to zero (m=8).

Supplementary Note 2:

Control analyses for Experiment 2

We performed a series of control analyses to ensure the results of Experiment 2 were robust (Supplementary Figure 8). In particular, we verified that there was no significant difference between Switch and Reference groups in motor and perceptual measures captured in the first portion of the paradigm (before switching), which was the same for both groups. As expected, step length asymmetry in overground and treadmill baselines and in the catch trial was not significantly different between groups (Switch – Reference SLA in OG baseline = 0.0050 [-0.0175, 0.0267], TM baseline = -0.0055 [-0.0328, 0.0203], catch = -0.0522 [-0.3910, 0.2817], mean [CI]); Similarly, there was no significant difference in perceptual bias in baseline or immediately post-adaptation (Switch – Reference bias in baseline = -0.0105 [-0.0333, 0.0118] m/s, post-adaptation = -0.0035 [-0.1455, 0.1430] m/s). We also found no significant difference between groups in either motor or perceptual generalizations, respectively measured as motor overground transfer (Supplementary Figure 8c) and perceptual treadmill decay (Supplementary Figure 8d; Switch – Reference motor overground transfer = -15.37% [-35.65, 1.56]%, perceptual treadmill decay = -9.04% [-29.33, 10.76]%). In line with results of Experiment 1, we also found that generalization in both domains was partial for both groups (motor overground transfer for Switch = 9.42% [6.50, 12.20]%, Reference = 24.79% [8.35, 44.91]%, perceptual treadmill decay for Switch = 63.97% [48.37, 77.14]%, Reference = 73.00% [58.29, 85.99]%; Supplementary Figure 8c-d). Note that we could not compute the motor treadmill decay measure for Experiment 2 because there are no post-adaptation tied-belt treadmill blocks.

Supplementary Tables

Supplementary Table 1. Overground aftereffects in the advancement of leg segments and joint angles at heel strike and toe off events. Aftereffect is computed as the average of the first 5 strides in the post-adaptation block, minus the baseline average. Data is reported as group mean [CI].

		slow heel strike	fast toe off	fast heel strike	slow toe off
joint angles (deg)	slow knee angle	***8.8771 [5.1571, 13.9720]***	***2.4474 [1.3257, 3.4564]***	0.3009 [-1.0756, 1.5996]	-1.0680 [-2.4144, 0.2735]
	slow hip angle	0.5869 [-1.8202, 3.1241]	***-5.3072 [-6.5908, -4.1733]***	-0.5045 [-1.9012, 1.2082]	0.6192 [-1.0556, 2.9683]
	fast hip angle	***5.3979 [3.8990, 7.1091]***	***4.5459 [2.8987, 6.7686]***	-0.8668 [-1.8865, 0.1364]	-1.0046 [-2.9118, 0.7720]
	fast knee angle	-0.3910 [-1.8346, 1.0053]	***2.2048 [1.3118, 3.2163]***	1.4634 [-0.1051, 3.4676]	-1.7581 [-3.2755, 0.1056]
segment advancement (m)	slow shank	***-0.0416 [-0.0585, -0.0237]***	***-0.0494 [-0.0601, -0.0379]***	0.0074 [-0.0078, 0.0199]	-0.0079 [-0.0181, 0.0005]
	slow thigh	0.0032 [-0.0074, 0.0134]	***-0.0318 [-0.0409, -0.0229]***	0.0055 [-0.0066, 0.0154]	0.0001 [-0.0156, 0.0117]
	pelvis	-0.0086 [-0.0173, 0.0007]	-0.0055 [-0.0104, 0.0004]	0.0022 [-0.0048, 0.0091]	0.0039 [-0.0022, 0.0088]
	fast thigh	***-0.0385 [-0.0498, -0.0278]***	***-0.0307 [-0.0469, -0.0184]***	-0.0056 [-0.0120, 0.0005]	-0.0071 [-0.0193, 0.0040]
	fast shank	***-0.0403 [-0.0524, -0.0276]***	***-0.0152 [-0.0243, -0.0077]***	-0.0151 [-0.0367, 0.0005]	0.0070 [-0.0066, 0.0197]

Note: Bold italic values represent aftereffects significantly different from zero.

Supplementary Table 2. Overground aftereffects in support times. Aftereffect is computed as the average of the first 5 strides in the post-adaptation block, minus the baseline average. Data is reported as group mean [CI].

**Support time (s)**	**Mean [CI] of change**
fast double support *(shs to fto)*	***0.0917 [0.0742, 0.1106]***
slow single support *(fto to fhs)*	-0.0096 [-0.0230, 0.0039]
slow double support *(fhs to sto)*	-0.0062 [-0.0262, 0.0141]
fast single support *(sto to shs)*	0.0017 [-0.0119, 0.0142]

Note: Bold italic values represent aftereffects significantly different from zero.

Supplementary Table 3. Overground aftereffects in peak flexion and extension of the hip and knee. Aftereffect is computed as the average of the first 5 strides in the post-adaptation block, minus the baseline average. Data is reported as group mean [CI].

	Joint angle (deg)	Mean [CI] of change
Slow leg	Peak knee flexion	-1.1998 [-3.0655, 0.6497]
	Peak knee extension	***-5.2376 [-7.1574, -3.2710]***
	Peak hip flexion	0.4719 [-1.0654, 1.9006]
	Peak hip extension	-0.1957 [-1.8466, 1.1026]
Fast leg	Peak knee flexion	***-2.7164 [-5.7152, -0.3951]***
	Peak knee extension	***-1.2569 [-2.7257, -0.0765]***
	Peak hip flexion	-0.2107 [-1.8423, 1.3373]
	Peak hip extension	***-3.4684 [-5.1900, -2.2463]***

Note: Bold italic values represent aftereffects significantly different from zero.

Supplementary Table 4. Overground walking speed of individual participants in all groups of Experiment 1. Mean [CI] for each participant's baseline and post-adaptation blocks. Individual participant ID is reported on the left column, and all speed values are reported in m/s.

	Short Preferred		Long Preferred		Short Slow	
	**Baseline**	**Post-adapt**	**Baseline**	**Post-adapt**	**Baseline**	**Post-adapt**
1	***0.885 [0.703, 0.977]***	***1.063 [0.920, 1.186]***	***1.298 [1.221, 1.355]***	***1.266 [1.154, 1.377]***	0.503 [0.464, 0.575]	0.496 [0.439, 0.581]
2	***1.067 [0.851, 1.195]***	***1.046 [0.723, 1.213]***	***0.834 [0.759, 0.876]***	***0.793 [0.624, 0.912]***	0.508 [0.480, 0.582]	0.519 [0.475, 0.591]
3	***1.122 [1.081, 1.161]***	***0.979 [0.808, 1.085]***	***0.991 [0.678, 1.048]***	***1.006 [0.870, 1.090]***	0.492 [0.412, 0.531]	0.468 [0.408, 0.515]
4	***0.788 [0.701, 0.926]***	***0.743 [0.658, 0.840]***	***1.088 [1.012, 1.211]***	***1.120 [0.971, 1.249]***	0.479 [0.404, 0.531]	0.519 [0.386, 0.662]
5	***1.217 [1.161, 1.265]***	***1.115 [0.928, 1.211]***	***1.096 [0.974, 1.174]***	***1.214 [0.994, 1.362]***	0.549 [0.509, 0.598]	0.527 [0.391, 0.697]
6	***0.869 [0.816, 0.942]***	***0.909 [0.723, 1.016]***	***0.986 [0.926, 1.052]***	***1.056 [0.970, 1.143]***	0.557 [0.492, 0.622]	0.546 [0.463, 0.622]
7	***1.134 [0.926, 1.215]***	***1.087 [0.834, 1.144]***	***1.043 [0.996, 1.093]***	***1.114 [1.038, 1.254]***	0.478 [0.402, 0.576]	0.457 [0.405, 0.501]
8	***0.969 [0.866, 1.115]***	***0.887 [0.735, 1.114]***	***1.148 [1.017, 1.220]***	***1.166 [1.017, 1.419]***	0.458 [0.390, 0.508]	0.470 [0.395, 0.582]
9	***1.169 [1.087, 1.239]***	***1.197 [0.978, 1.328]***	***1.116 [1.035, 1.201]***	***1.094 [1.003, 1.184]***	0.450 [0.431, 0.467]	0.474 [0.431, 0.515]
10	***1.201 [1.046, 1.310]***	***1.082 [0.637, 1.292]***	***1.045 [0.988, 1.099]***	***0.931 [0.850, 1.019]***	0.508 [0.456, 0.541]	0.509 [0.457, 0.554]

Note: Bold italic values represent speeds for which the CI does not overlap the 0.45-0.55m/s range (significantly faster than 0.55m/s or significantly slower than 0.45m/s).

Supplementary Table 5. Demographics of participants. Group (column 1), self-reported sex (column 2), age (column 3), and leg dominance ("leg you would use to kick a ball"; column 4).

Group	Sex	Age	Leg dominance
Short Preferred	M	24	R
	F	26	L
	M	20	R
	F	18	R
	F	18	R
	F	20	R
	F	29	R
	M	28	R
	F	25	R
	F	25	R
Long Preferred	F	26	R
	M	18	R
	F	20	R
	F	25	R
	F	28	R
	M	21	L
	M	24	R
	M	23	R
	F	32	R
	M	23	R
Short Slow	M	32	L
	F	29	R
	F	23	R
	F	31	L
	M	24	R
	F	25	R
	F	34	R
	F	19	R
	F	35	R
	M	25	R
Switch Slow	F	24	R
	F	24	R
	F	26	R
	M	29	R
	F	25	R
	F	21	R
	F	27	R
	F	19	R
	F	29	R
	F	20	R
Switch control	M	29	R
	F	27	R
	M	31	R
	M	19	L
	F	27	R
	F	23	R
	F	20	R
	M	25	R
	F	29	R
	F	24	R

Supplementary References

1. Kambic, R. E., Roemmich, R. T. & Bastian, A. J. Joint-level coordination patterns for split-belt walking across different speed ratios. *J. Neurophysiol.* **129**, 969–983 (2023).
2. Reisman, D. S., Block, H. J. & Bastian, A. J. Interlimb coordination during locomotion: what can be adapted and stored? *J. Neurophysiol.* **94**, 2403–2415 (2005).
3. Mariscal, D. M., Vasudevan, E. V. L., Malone, L. A., Torres-Oviedo, G. & Bastian, A. J. Context-Specificity of Locomotor Learning Is Developed during Childhood. *eNeuro* **9**, (2022).
4. Mariscal, D. M., Iturralde, P. A. & Torres-Oviedo, G. Altering attention to split-belt walking increases the generalization of motor memories across walking contexts. *J. Neurophysiol.* **123**, 1838–1848 (2020).
5. Sombric, C. J. & Torres-Oviedo, G. Cognitive and Motor Perseveration Are Associated in Older Adults. *Front. Aging Neurosci.* **13**, 610359 (2021).
6. Sombric, C. J., Harker, H. M., Sparto, P. J. & Torres-Oviedo, G. Explicit action switching interferes with the context-specificity of motor memories in older adults. *Front. Aging Neurosci.* **9**, 40 (2017).
7. Torres-Oviedo, G. & Bastian, A. J. Natural error patterns enable transfer of motor learning to novel contexts. *J. Neurophysiol.* **107**, 346–356 (2012).
8. Eikema, D. J. A. *et al.* Optic flow improves adaptability of spatiotemporal characteristics during split-belt locomotor adaptation with tactile stimulation. *Exp. Brain Res.* **234**, 511–522 (2016).
9. Hamzey, R. J., Kirk, E. M. & Vasudevan, E. V. L. L. Gait speed influences aftereffect size following locomotor adaptation, but only in certain environments. *Exp. Brain Res.* **234**, 1479–1490 (2016).
10. Kim, D., Desrochers, P. C., Lewis, C. L. & Gill, S. V. Effects of obesity on adaptation transfer from treadmill to over-ground walking. *Appl. Sci.* **11**, 2108 (2021).
11. Mukherjee, M. *et al.* Plantar tactile perturbations enhance transfer of split-belt locomotor adaptation. *Exp. Brain Res.* **233**, 3005–3012 (2015).
12. Reisman, D. S., Wityk, R., Silver, K. & Bastian, A. J. Split-belt treadmill adaptation transfers to overground walking in persons poststroke. *Neurorehabil. Neural Repair* **23**, 735–744 (2009).
13. Aucie, Y., Harket, H. M., Sombric, C. J. & Torres-Oviedo, G. Older adults generalize their movements across walking contexts more than young during gradual and abrupt split-belt walking. *bioRxiv* 2021.08.06.455403 (2021). doi:10.1101/2021.08.06.455403
14. Huynh, K. V., Sarmento, C. H., Roemmich, R. T., Stegemöller, E. L. & Hass, C. J. Comparing aftereffects after split-belt treadmill walking and unilateral stepping. *Med. Sci. Sports Exerc.* **46**, 1392–1399 (2014).

Supplementary Figures

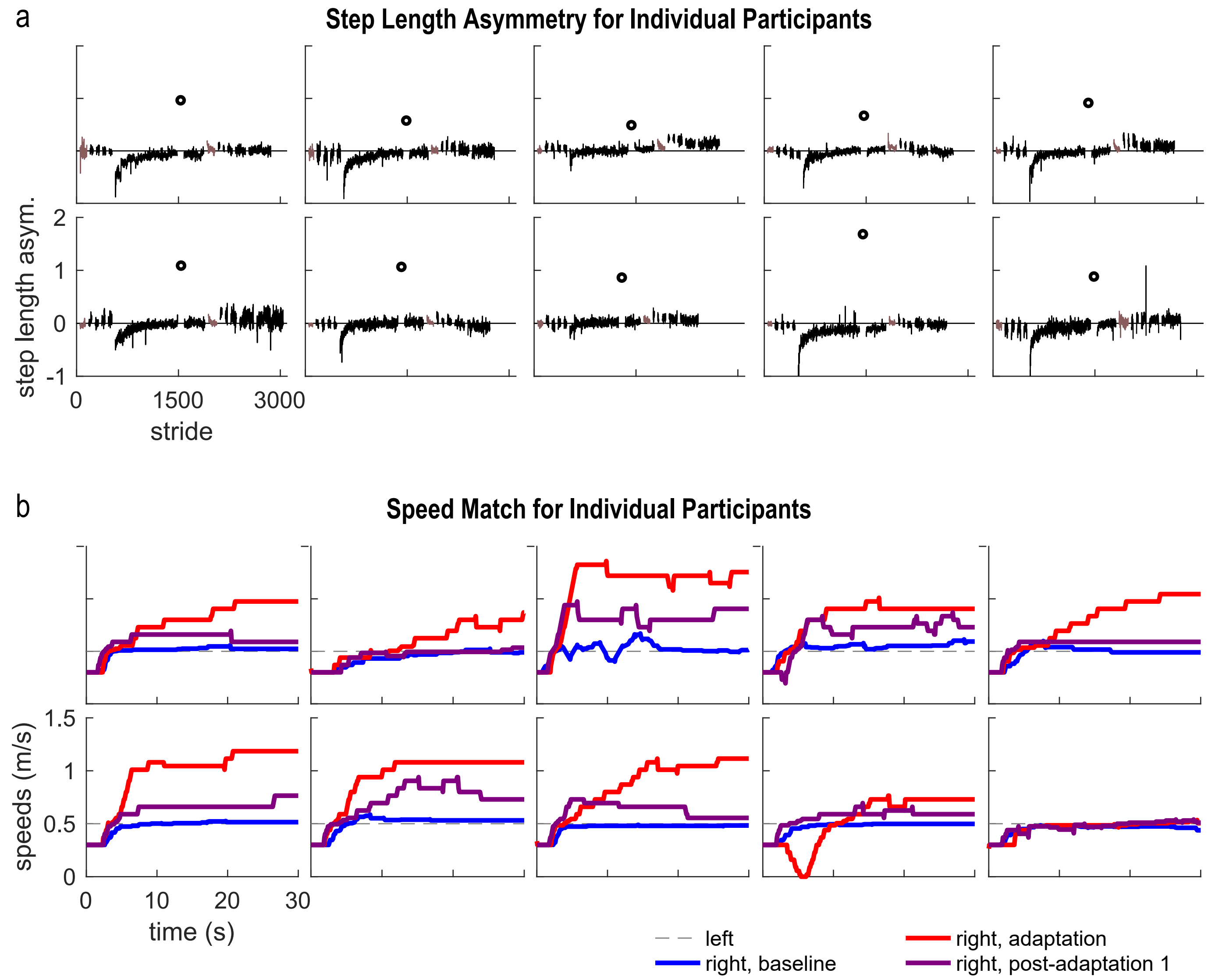

Supplementary Figure 1. Individual participant data for Experiment 1A. (a) Step length asymmetry timecourse. The open circle depicts the catch trial (average of first 5 strides). **(b)** Belt speeds during selected iterations of the speed match task: baseline (blue, average of three iterations), adaptation (red), and first post-adaptation iteration (purple).

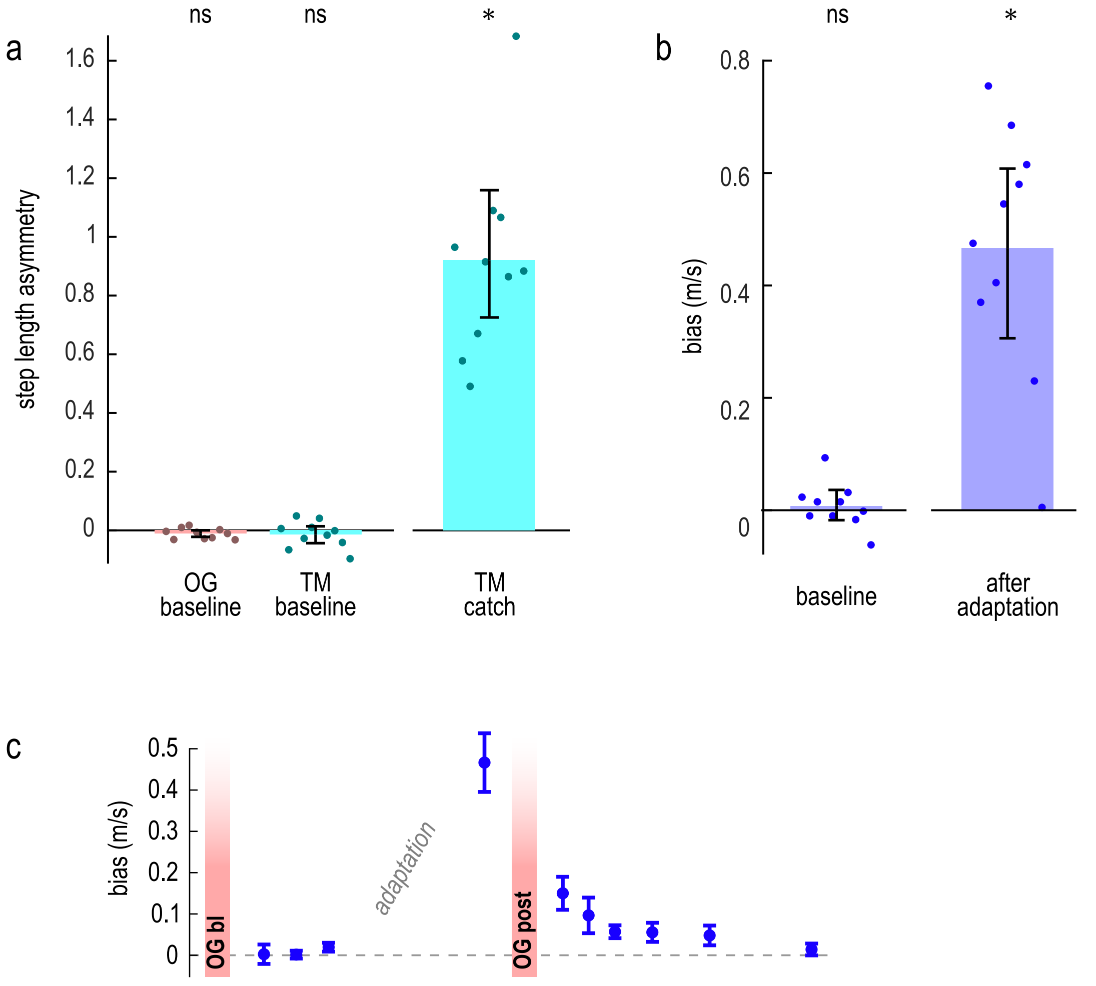

Supplementary Figure 2. Additional results for Experiment 1A. (a) Step length asymmetry in the overground baseline, treadmill baseline, and treadmill catch trial epochs. **(b)** Perceptual bias for the speed match tasks performed in baseline (average of three iterations) and immediately after adaptation). For both panel (a) and (b), bars and error bars depict group mean ± CI, dots depict individual participants, and asterisks indicate the measure is significantly different from zero. **(c)** Perceptual bias for all iterations of the speed match task (group mean ± SE).

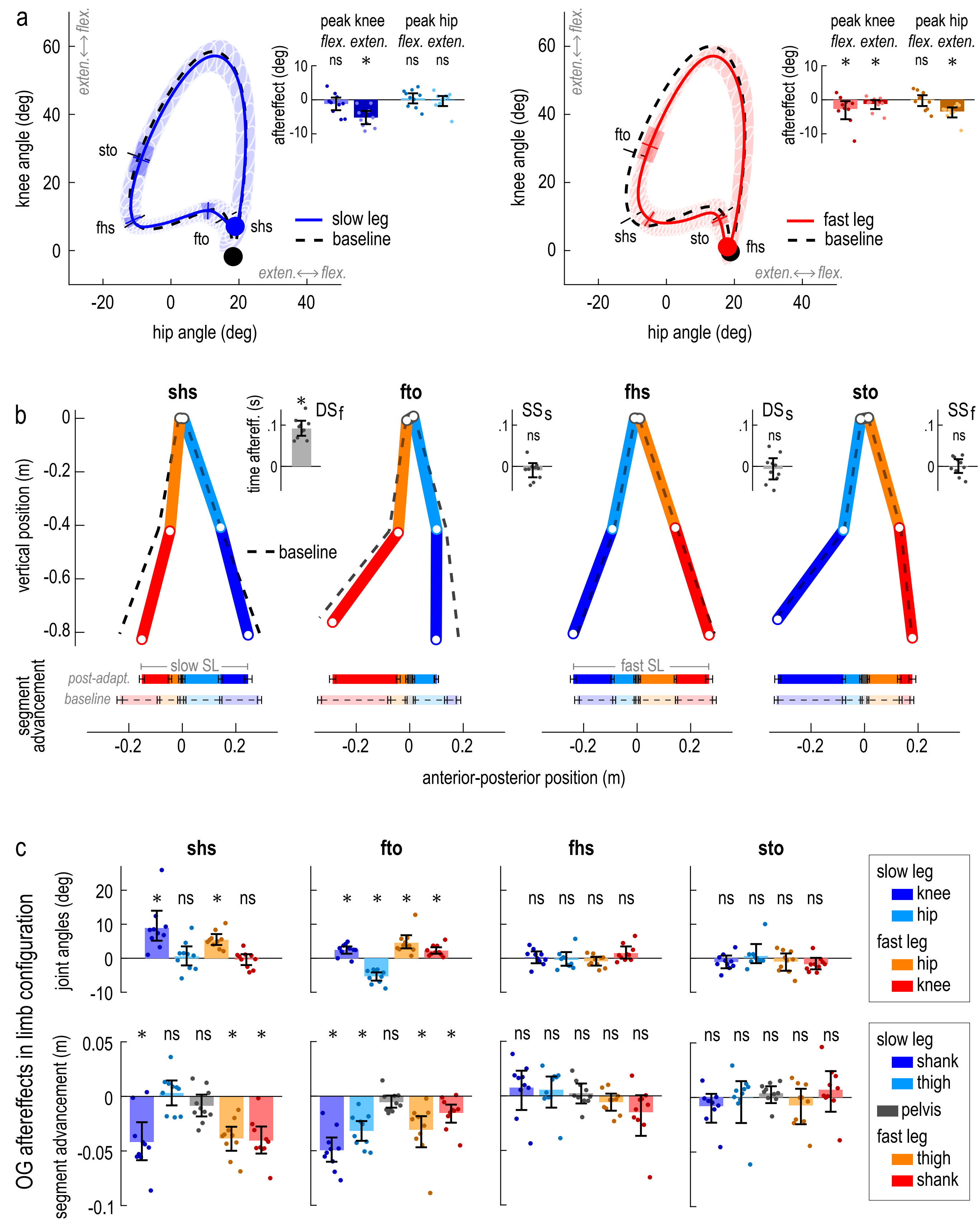

Supplementary Figure 3. Overground aftereffects in joint kinematic patterns, segment configuration and temporal parameters. (a) Knee versus hip angle traces for a stride cycle. Blue and red trace depict group mean for the slow and fast leg in post-adaptation; baseline is depicted with a dashed black line on the same plot (group mean). Group standard deviation ellipses for each interpolated time point in the stride cycle are shaded (white traces are included every 2% of the stride cycle). Data was interpolated for each stride and pre-averaged for the strides in the epoch (all baseline strides, first 5 post-adaptation strides) for each participant. The start of the stride is depicted with a circle (shs or fhs), and the timing for the other stride cycle events is depicted with lines perpendicular to the angle trace (group mean); the shade for the post-adaptation events represent group standard deviation. **Insets:** aftereffect in peak flexion and extension of the knee and hip angles (post-adaptation minus baseline). Bars and error bars depict group mean ± CI, dots depict individual participants, and asterisks indicate significant aftereffects. **(b)** Configuration of leg segments at each event in the stride cycle, in baseline (dashed black) and post-adaptation (color). Position of the markers (white circles) is averaged across participants. **Bottom:** segment advancement, consisting of the projection of each segment on the anterior-posterior axis. **Inset:** aftereffects (post-adaptation minus baseline) in the time between stride cycle events (fast double support, slow single support, slow double support, fast single support). Bars and error bars depict group mean ± CI, dots depict individual participants, and asterisks indicate significant aftereffects. **(c)** Aftereffects (post-adaptation minus baseline) in joint angles (top) and segment advancement (bottom) at each stride cycle event. Bars and error bars depict group mean ± CI, dots depict individual participants, and asterisks indicate significant aftereffects.

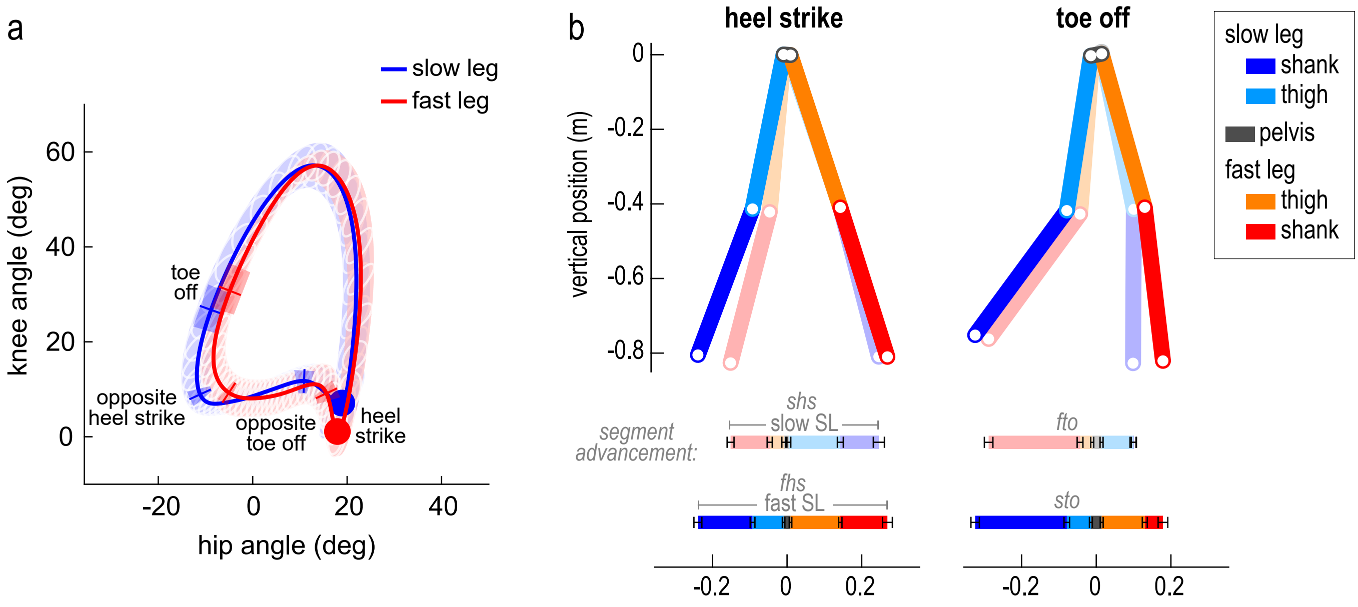

Supplementary Figure 4. Overground aftereffects in joint kinematic patterns and segment configuration, with legs overlayed to highlight asymmetries. Data is depicted as in Fig. S3. **(a)** Knee versus hip angle traces for a stride cycle. Blue and red trace depict group mean for the slow and fast leg in post-adaptation. Group standard deviation ellipses for each interpolated time point in the stride cycle are shaded (white traces are included every 2% of the stride cycle). Data was interpolated for each stride and pre-averaged for the strides in the epoch (all baseline strides, first 5 post-adaptation strides) for each participant. The start of the stride is depicted with a circle (shs or fhs), and the timing for the other stride cycle events is depicted with lines perpendicular to the angle trace (group mean); the shade for the post-adaptation events represent group standard deviation. **(b)** Configuration of leg segments at heel strike and toe off events in the stride cycle (fhs and sto are in front, shs and fto are in the back and transparent). Position of the markers (white circles) is averaged across participants. **Bottom:** segment advancement, consisting of the projection of each segment on the anterior-posterior axis.

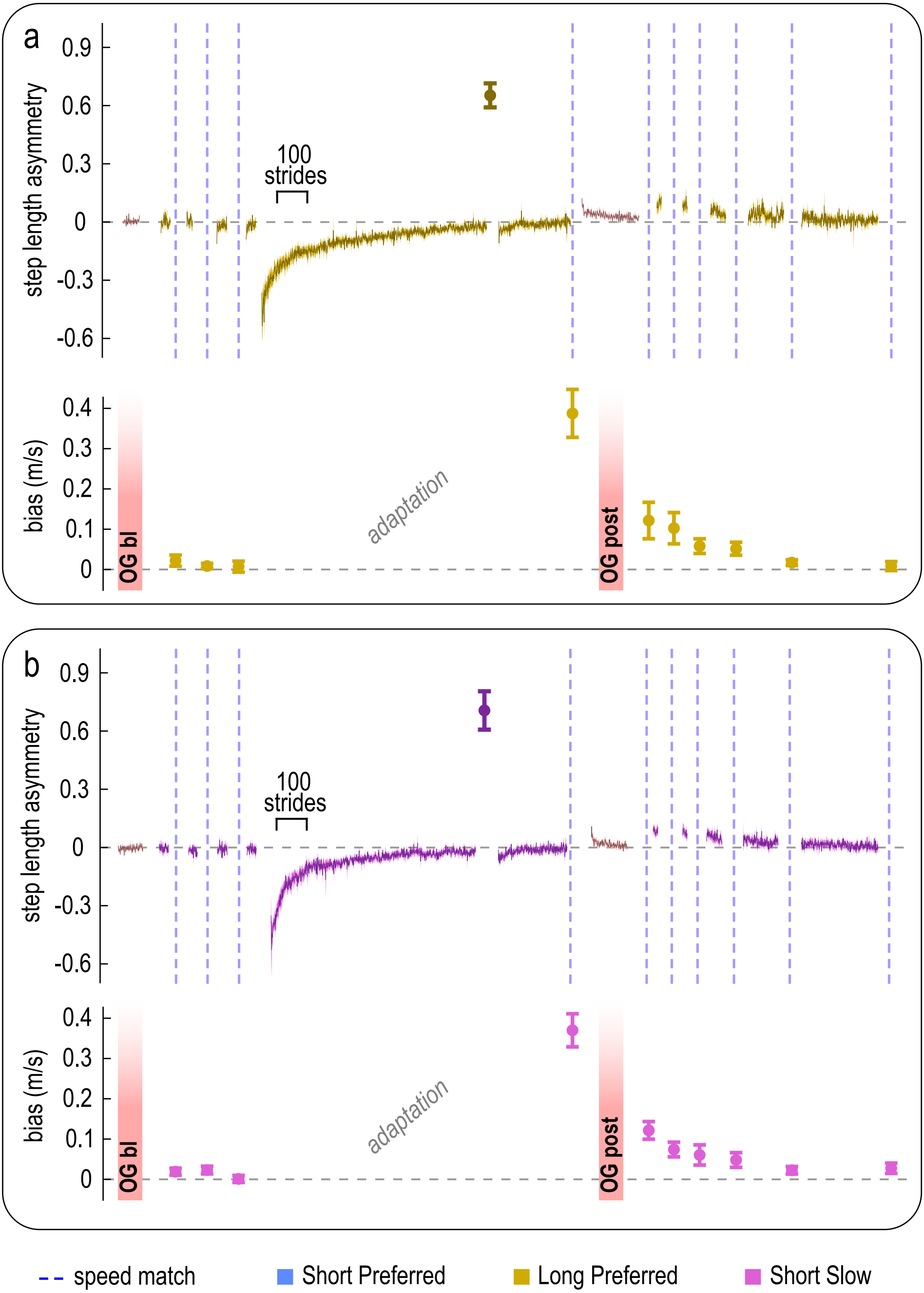

Supplementary Figure 5. Motor and perceptual timeseries results for Long Preferred (a) and Short Slow (b) groups of Experiment 1B. (a-b) top: Step length asymmetry time course (group mean ± SE). The length of each block is truncated to the participant with the fewest strides. Vertical dashed lines represent iterations of the speed match task. **(a-b) bottom:** Perceptual bias for all iterations of the speed match task (group mean ± SE); the position matches that of the respective vertical lines in the plot above it.

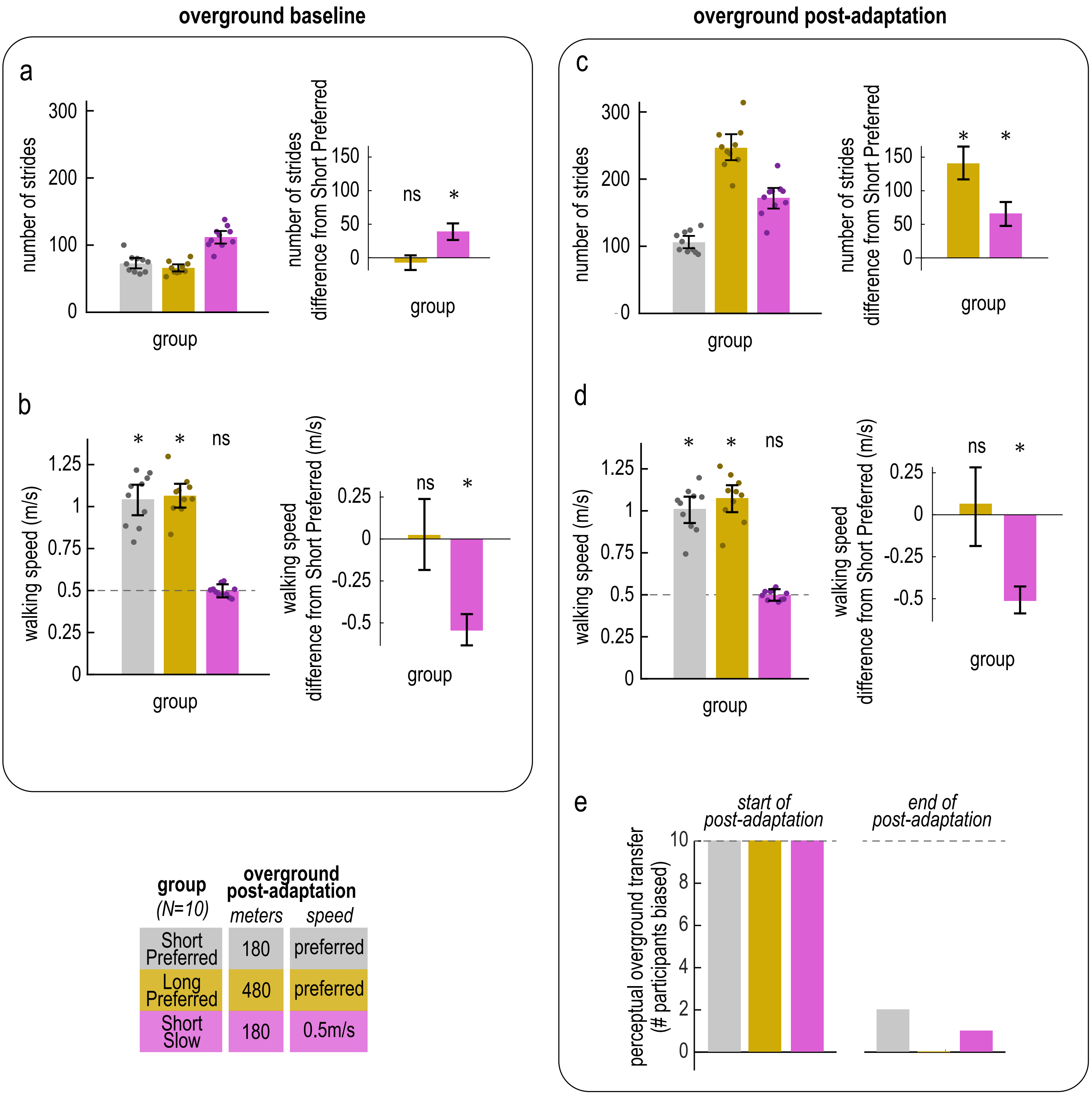

Supplementary Figure 6. Results to confirm the manipulations of Experiment 1B during overground baseline (left panel, a-b) and overground post-adaptation (right panel, c-e). (a, c) left: Number of strides in overground baseline (a) and overground post-adaptation (c) for Short Preferred (gray), Long Preferred (yellow) and Short Slow (pink). **(a, c) right:** Difference in number of strides between Long Preferred and Short Preferred (yellow) and between Short Slow and Short Preferred (pink). Asterisks indicate a significant difference between groups. **(b, d) left:** Overground walking speed in overground baseline (b) and overground post-adaptation (d) for all groups (colors are as in (a) and (c)). Asterisks indicate the measure is significantly different from 0.5m/s (left). **(b, d) right:** Difference in overground walking speed relative to Short Preferred (colors are as in (a) and (c)). Asterisks indicate the measure is significantly different between the groups. For panels (a-d), bars and error bars depict group mean ± CI, and dots depict individual participants. **(e)** Number of participants with a perceptual bias at the start (left) and at the end (right) of overground post-adaptation, for all groups (colors are as in (a-d)).

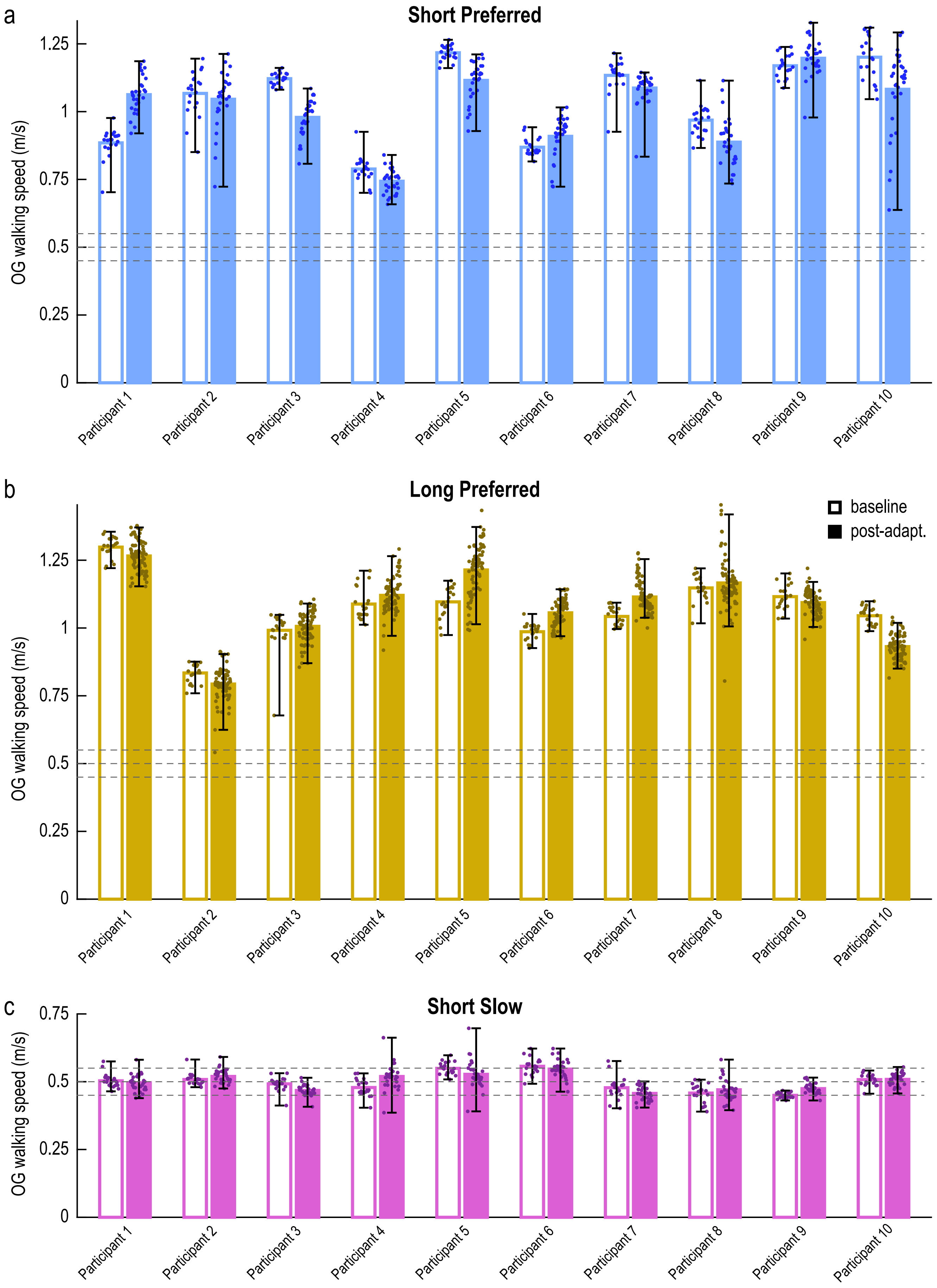

Supplementary Figure 7. Overground walking speed for individual participants in Experiment 1. Bars and error bars depict mean ± CI computed within-participant for each phase; baseline is depicted with open bars and post-adaptation with filled bars. Dots depict the speed of individual passes. Horizontal dashed lines are indicative of the 0.45-0.55m/s target speed range for the Short Slow group.

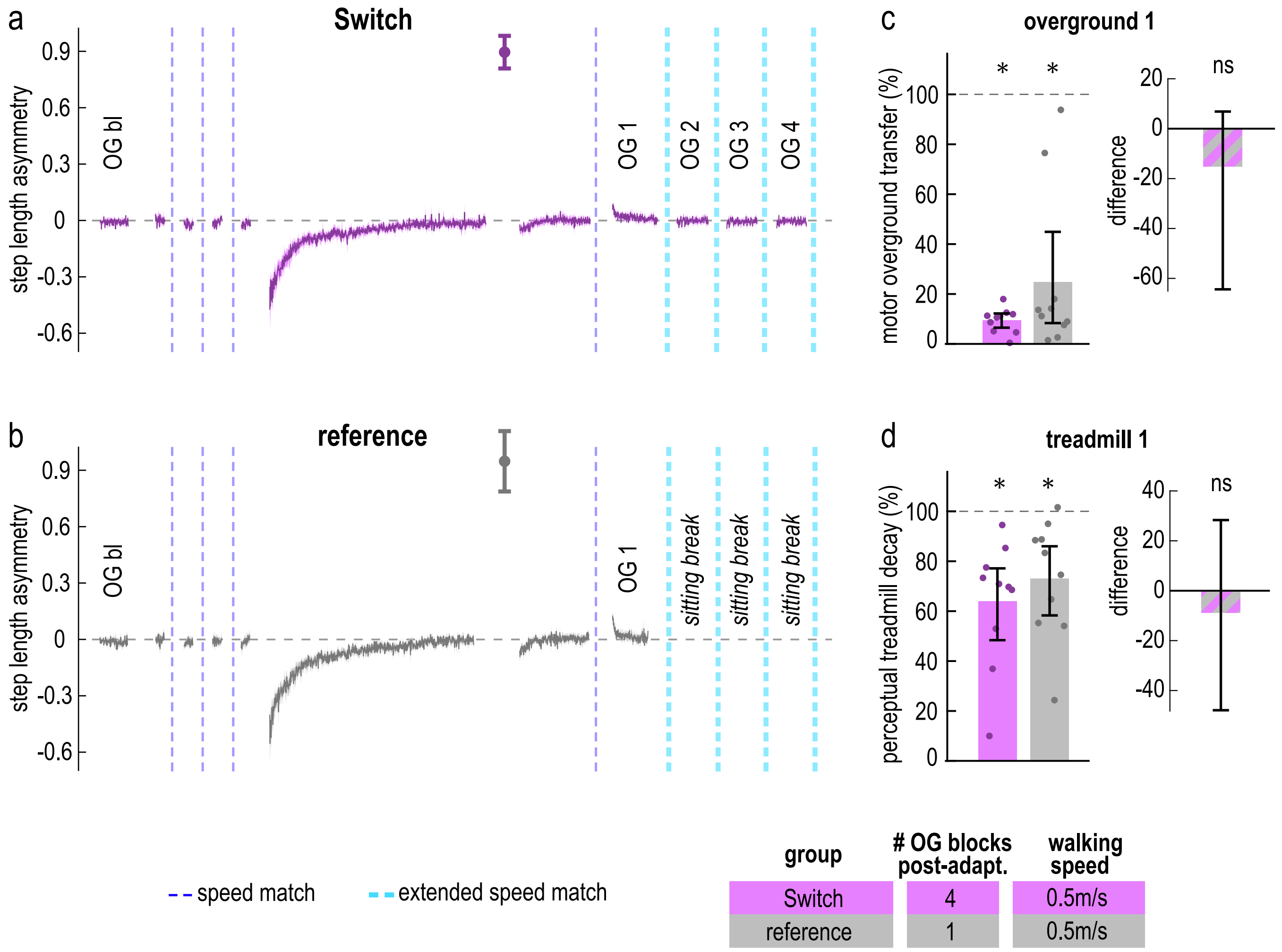

Supplementary Figure 8. Additional results for Experiment 2. (a-b) Step length asymmetry time course (group mean ± SE). The length of each block is truncated to the participant with the fewest strides. Vertical dashed lines represent iterations of the speed match task. **(c)** Motor overground transfer (left), and difference in the measure between the groups (right). **(d)** Perceptual treadmill decay (left), and difference in the measure between the groups (right). For both panel (c) and (d), bars and error bars depict group mean ± CI, and dots depict individual participants. Asterisks indicate the measure is significantly different from 0% and 100% (left) or between the groups (right). In all plots, the Switch group is depicted in purple, the reference group in gray, and Switch - references differences are purple-gray hatched.

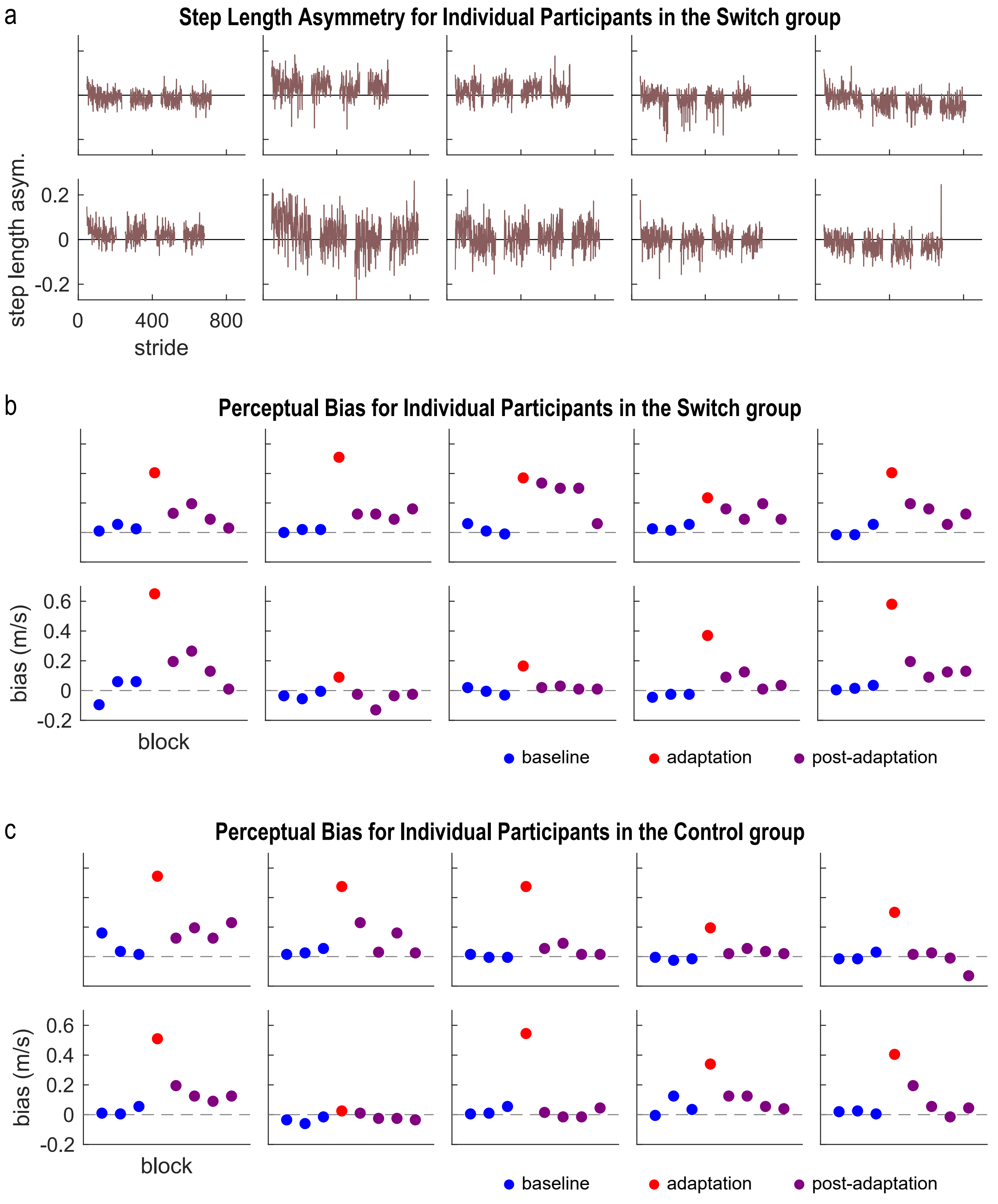

Supplementary Figure 9. Individual participant data for Experiment 2. (a) Step length asymmetry post-adaptation timecourse for participants in the Switch group. **(b-c)** Perceptual bias (right belt speed - left belt speed) for all iterations of the speed match task (blue = baseline, red = adaptation, purple = post-adaptation), for the Switch (b) and Control (c) groups.
