# Supplementary material for: Understanding mechanisms of generalization following locomotor adaptation
Source: NPJ Sci Learn. 2024 Jul 23;9:48. doi: 10.1038/s41539-024-00258-2 (PMC11266392; doi:10.1038/s41539-024-00258-2)
Supplement: Supplementary file 2 — Reporting Summary [file 41539_2024_258_MOESM2_ESM.pdf]

nature portfolio

Corresponding author(s): DBPR NPJSCILEARN-01003R

Last updated by author(s): Jun 16, 2024

Reporting Summary

Nature Portfolio wishes to improve the reproducibility of the work that we publish. This form provides structure for consistency and transparency in reporting. For further information on Nature Portfolio policies, see our Editorial Policies and the Editorial Policy Checklist.

Statistics

For all statistical analyses, confirm that the following items are present in the figure legend, table legend, main text, or Methods section.

n/a	Confirmed	
☐	☒	The exact sample size (*n*) for each experimental group/condition, given as a discrete number and unit of measurement
☐	☒	A statement on whether measurements were taken from distinct samples or whether the same sample was measured repeatedly
☐	☒	The statistical test(s) used AND whether they are one- or two-sided<br>*Only common tests should be described solely by name; describe more complex techniques in the Methods section.*
☐	☒	A description of all covariates tested
☐	☒	A description of any assumptions or corrections, such as tests of normality and adjustment for multiple comparisons
☐	☒	A full description of the statistical parameters including central tendency (e.g. means) or other basic estimates (e.g. regression coefficient) AND variation (e.g. standard deviation) or associated estimates of uncertainty (e.g. confidence intervals)
☐	☒	For null hypothesis testing, the test statistic (e.g. *F*, *t*, *r*) with confidence intervals, effect sizes, degrees of freedom and *P* value noted<br>*Give P values as exact values whenever suitable.*
☒	☐	For Bayesian analysis, information on the choice of priors and Markov chain Monte Carlo settings
☒	☐	For hierarchical and complex designs, identification of the appropriate level for tests and full reporting of outcomes
☒	☐	Estimates of effect sizes (e.g. Cohen's *d*, Pearson's *r*), indicating how they were calculated

Our web collection on statistics for biologists contains articles on many of the points above.

Software and code

Policy information about availability of computer code

Data collection	Vicon Motion Systems software
Data analysis	MATLAB custom algorithms, deposited in repository, link provided in paper

For manuscripts utilizing custom algorithms or software that are central to the research but not yet described in published literature, software must be made available to editors and reviewers. We strongly encourage code deposition in a community repository (e.g. GitHub). See the Nature Portfolio guidelines for submitting code & software for further information.

Data

Policy information about availability of data

All manuscripts must include a data availability statement. This statement should provide the following information, where applicable:

- Accession codes, unique identifiers, or web links for publicly available datasets
- A description of any restrictions on data availability
- For clinical datasets or third party data, please ensure that the statement adheres to our policy

Data and code availability

Note to reviewers: the dataset cited below is currently “private for peer review”, and can be accessed by the alternative “reviewer URL” provided by the Dryad database: https://datadryad.org/stash/share/p4al_DkzY7aunqlBB2f3SWEL6f0abiolqTVbeA4ujbY.

The datasets generated and analyzed during the current study, and the code used for the analyses, are available in the Dryad repository: Rossi, Roemmich, Bastian (2023). “Dataset for Understanding mechanisms of generalization following locomotor adaptation”. https://doi.org/10.5061/dryad.g4f4qrfwc.

Research involving human participants, their data, or biological material

Policy information about studies with human participants or human data. See also policy information about sex, gender (identity/presentation), and sexual orientation and race, ethnicity and racism.

Reporting on sex and gender	Sex was collected based on self-reporting. Sex and gender were not considered in the study design because there is no hypothesized role of these data in the system we study. Disaggregated data is provided. We enrolled 34 female and 16 male participants.
Reporting on race, ethnicity, or other socially relevant groupings	No socially constructed or socially relevant categorization variables were used in the manuscript. These variables are not hypothesized to play a role in the system we study.
Population characteristics	The mean ± SD age was 25.0 ± 4.3 years; the range was 18-35 years. Other characteristics are not relevant.
Recruitment	Participants were recruited through a university email list. Socially related variables are not thought to affect the basic biological process described in our study, so we do not think this factor is likely to impact our results.
Ethics oversight	Johns Hopkins Institutional Review Board

Note that full information on the approval of the study protocol must also be provided in the manuscript.

Field-specific reporting

Please select the one below that is the best fit for your research. If you are not sure, read the appropriate sections before making your selection.

☐ Life sciences ☒ Behavioural & social sciences ☐ Ecological, evolutionary & environmental sciences

For a reference copy of the document with all sections, see nature.com/documents/nr-reporting-summary-flat.pdf

Behavioural & social sciences study design

All studies must disclose on these points even when the disclosure is negative.

Study description	Quantitative experimental. We collected kinematic data regarding the movement of the joints of the legs during human walking.
Research sample	The mean ± SD age was 25.0 ± 4.3 years; the range was 18-35 years. Participants were recruited through a university email list at Johns Hopkins University (sample included Johns Hopkins Undergraduate students, graduate students, staff, as well as participants not affiliated with Johns Hopkins). Age may affect the results; different studies have focused on younger or older populations and this is beyond the scope of our study. Socially related variables are not thought to affect the basic biological process described in our study, so we do not think this factor is likely to impact our results.
Sampling strategy	We report the analysis for determining sample size in the manuscript. We used G-Power software to analyze existing data and estimated the number of participants needed for having at least 80% power in our primary measures of interest. We tested groups sequentially and participants were assigned to different groups based on the order they enrolled. Because all of our analysis are basic science and not affected by social variables, we do not think that this affected the results.
Data collection	For treadmill portion, participants walked on a Motek (Amsterdam, NL) split-belt treadmill with the belt speeds controlled by the D-Flow environment (Motek, Amsterdam, NL), kinematic data was recorded at 100Hz using a Vicon motion capture system (Oxford, UK). For overground portions, participants walked on a six-meter-long walkway, and data was collected using Zeno Walkway (ProtoKinetics, Havertown, PA), or Vicon motion capture system (Oxford, UK).
Timing	Short Preferred: April 2019 - June 2019. Long Preferred: April 2019 - June 2019. Short Slow: December 2020 - April 2021. Switch Group: April 2021 - May 2021. Reference Group: November 2021 - January 2022.
Data exclusions	7 participants were excluded for equipment issues or for not following directions.
Non-participation	1 participant dropped out after the first baseline block.
Randomization	Participants were allocated to groups sequentially. Socially related variables are not thought to affect the basic biological process described in our study, so we do not think this factor is likely to impact our results.

Reporting for specific materials, systems and methods

We require information from authors about some types of materials, experimental systems and methods used in many studies. Here, indicate whether each material, system or method listed is relevant to your study. If you are not sure if a list item applies to your research, read the appropriate section before selecting a response.

Materials & experimental systems

n/a	Involved in the study
☒	☐ Antibodies
☒	☐ Eukaryotic cell lines
☒	☐ Palaeontology and archaeology
☒	☐ Animals and other organisms
☒	☐ Clinical data
☒	☐ Dual use research of concern
☒	☐ Plants

Methods

n/a	Involved in the study
☒	☐ ChIP-seq
☒	☐ Flow cytometry
☒	☐ MRI-based neuroimaging

Plants

Seed stocks	n/a
Novel plant genotypes	n/a
Authentication	n/a
